# Sex Differences in the Adult Human Brain: Evidence from 5216 UK Biobank Participants

**DOI:** 10.1093/cercor/bhy109

**Published:** 2018-05-16

**Authors:** Stuart J Ritchie, Simon R Cox, Xueyi Shen, Michael V Lombardo, Lianne M Reus, Clara Alloza, Mathew A Harris, Helen L Alderson, Stuart Hunter, Emma Neilson, David C M Liewald, Bonnie Auyeung, Heather C Whalley, Stephen M Lawrie, Catharine R Gale, Mark E Bastin, Andrew M McIntosh, Ian J Deary

**Affiliations:** 1 Department of Psychology, The University of Edinburgh, Edinburgh, UK; 2 Centre for Cognitive Ageing and Cognitive Epidemiology, The University of Edinburgh, Edinburgh, UK; 3 Division of Psychiatry, The University of Edinburgh, Edinburgh, UK; 4 Department of Psychology and Center for Applied Neuroscience, University of Cyprus, Nicosia, Cyprus; 5 Autism Research Centre, Department of Psychiatry, University of Cambridge, Cambridge, UK; 6 Department of Neurology and Alzheimer Centre, VU University Medical Centre, Amsterdam, The Netherlands; 7 Department of Psychiatry, Queen Margaret Hospital, Dunfermline, UK; 8 NHS Lothian, Edinburgh, UK; 9 MRC Lifecourse Epidemiology Unit, University of Southampton, Southampton, UK; 10 Brain Research Imaging Centre, The University of Edinburgh, Edinburgh, UK; 11 Centre for Clinical Brain Sciences, The University of Edinburgh, Edinburgh, UK

**Keywords:** brain volume, cortical thickness, fMRI, sex differences, surface area

## Abstract

Sex differences in the human brain are of interest for many reasons: for example, there are sex differences in the observed prevalence of psychiatric disorders and in some psychological traits that brain differences might help to explain. We report the largest single-sample study of structural and functional sex differences in the human brain (2750 female, 2466 male participants; mean age 61.7 years, range 44–77 years). Males had higher raw volumes, raw surface areas, and white matter fractional anisotropy; females had higher raw cortical thickness and higher white matter tract complexity. There was considerable distributional overlap between the sexes. Subregional differences were not fully attributable to differences in total volume, total surface area, mean cortical thickness, or height. There was generally greater male variance across the raw structural measures. Functional connectome organization showed stronger connectivity for males in unimodal sensorimotor cortices, and stronger connectivity for females in the default mode network. This large-scale study provides a foundation for attempts to understand the causes and consequences of sex differences in adult brain structure and function.

## Introduction

Sex differences have been of enduring biological interest (Darwin 1871), but our knowledge about their relevance to the human brain is surprisingly sparse. It has been noted by several researchers that the potential influences of sex are under-explored in neuroscientific research (Beery and Zucker 2011; Cahill 2006, 2017; Karp et al. 2017). A fuller understanding of morphological and functional differences between the brains of the human sexes might provide insight into why the observed prevalence of some psychiatric disorders differs substantially by sex (Rutter et al. 2003), and would assist in explaining several behavioral sex differences (Zell et al. 2015; Gur and Gur 2017). As biomedical research moves closer to the ideals of precision medicine (e.g., Collins and Varmus 2015), it is even more pressing that we have a more nuanced understanding of similarities and differences in brain structure and function across the sexes. Here, we report a study that characterizes multimodal sex differences in the adult human brain in the largest sample to date.

It is of particular importance to gain a more detailed picture of how the brains of males and females differ, because several psychiatric disorders and conditions differ in their prevalence between the sexes. For instance, rates of Alzheimer’s disease are higher in females than males, prompting a recent call for the prioritization of biomedical research into sex differences in measures relevant to this disorder (Mazure and Swendsen 2016). Females also show a higher prevalence of major depressive disorder (Rutter et al. 2003; Gobinath et al. 2017), whereas males display higher rates of disorders such as autism spectrum disorder (Baron-Cohen et al. 2011), schizophrenia (Aleman et al. 2003) and dyslexia (Arnett et al. 2017). Improving therapeutic strategies for these conditions will almost certainly require accurate quantitative estimates of where and how the sexes differ normatively.

Moreover, although many psychological sex differences are small (consistent with the “gender similarities hypothesis”; Hyde 2014), some behaviors and traits do show reliable and substantial differences. For instance, performance on mental rotation tasks (Maeda and Yoon 2013) and physical aggression (Archer 2004) are on average higher in males, whereas self-reported interest in people versus things (Su et al. 2009) and the personality traits of neuroticism (Schmitt et al. 2008) and agreeableness (Costa et al. 2001) are on average higher in females. A full explanation of these cognitive and behavioral phenomena might benefit from a better understanding of brain sex differences.

Our understanding of brain sex differences has been hampered by low statistical power in previous studies. Small-sample research has become a considerable concern in neuroscience research (Button et al. 2013; Nord et al. 2017), and the concern no less applies to research on sex differences. To illustrate this point, in the most recent meta-analysis of macrostructural sex differences in brain subregions (Ruigrok et al. 2014)—which revealed a complex pattern of differences, with both males and females showing larger brain volume depending on the brain substructure in question—studies that examined sex differences in specific subregions of interest (rather than in broad, overall measures) had a mean sample size of 130 participants (range = 28–465). Since the publication of that meta-analysis, some larger macrostructural studies have appeared, though they are either in younger participants only (Gur and Gur 2016; Gennatas et al. 2017; Wierenga et al. 2017) or somewhat limited in the number of brain measures they report (Jäncke et al. 2015). Adult macrostructural studies with a large scale—both in terms of sample size and in terms of brain regions analyzed—are required.

Beyond macrostructural measures, there may also be robust sex differences in measures of the brain’s white matter microstructure. Studies that have attempted to quantify sex differences in white matter microstructure with diffusion tensor MRI—which uses information about the movement of water molecules through the brain’s white matter tracts to produce measures such as fractional anisotropy (FA), which has been linked to variation in cognitive and health-related traits (Sundgren et al. 2004)—are rare and, where they exist, small in sample size (Kanaan et al. 2012; Dunst et al. 2014). Newer and more rarely-studied neurite orientation dispersion and density imaging (NODDI) measures can offer novel information on the microstructural bases of sex differences. NODDI treats the diffusion-weighted signal as a combination of 3 compartments, each with characteristics that differentially shape the diffusion probability function of water molecules: fast isotropic diffusion (e.g., cerebrospinal fluid), anisotropic hindered diffusion (e.g., extracellular water), and restricted anisotropic diffusion (e.g., intra-axonal compartments; Zhang et al. 2012). This relatively more complex parameterization of the water diffusion signal may also lead to lower susceptibility to partial volume effects, and can potentially yield less ambiguous estimates of underlying microstructure than more traditional measures (for instance, FA cannot resolve these different underlying tissue properties).

In addition to the above structural brain imaging measures, it is also of interest to investigate sex differences in brain function. Examinations of sex differences in resting-state functional connectivity—the functional measure used in the present study, which indexes the temporal relations between activation in anatomically-separate brain regions while the brain is at rest (that is, not completing any experimenter-directed task; van den Heuvel and Hulshoff Pol 2010)—have also shown substantial differences, for example within the default mode network (where females show stronger connectivity) and within sensorimotor and visual cortices (where males show stronger connectivity; Biswal et al. 2010). As has been noted (Scheinost et al. 2015), a better characterization of broad patterns, including sex differences, in relatively novel measures such as functional connectivity (and in the NODDI parameters described above) is of importance to establish a “baseline” upon which future studies of normal versus abnormal function can rely.

There is more to sex differences than averages: there are physical and psychological traits that tend to be more variable in males than females. The best-studied human phenotype in this context has been cognitive ability: almost universally, studies have found that males show greater variance in this trait (Deary et al. 2007a; Johnson et al. 2008; Lakin 2013; though see Iliescu et al. 2016). This has also been found for academic achievement test results (themselves a potential consequence of cognitive differences, which are known to predict later educational achievement; Deary et al. 2007b; Machin and Pekkarinen 2008; Lehre et al. 2009a, 2009b), other psychological characteristics such as personality (Borkenau et al. 2013), and a range of physical traits such as athletic performance (Olds et al. 2006), and both birth and adult weight (Lehre et al. 2009a). To our knowledge, only two prior studies have explicitly examined sex differences in the variability of brain structure (Wierenga et al. 2017; Lange et al. 1997), and no studies have done so in individuals older than 20 years. Here, we addressed this gap in the literature by testing the “greater male variability” hypothesis in the adult brain.

### The Present Study

To date, there exists no single, comprehensive, well-powered analysis of sex differences in mean and variance in the adult brain that covers structural, diffusion, and functional MRI measures. Here, we examine multimodal sex differences in adult human brain structural and functional organization in the largest and most definitive study to date, ensuring high levels of statistical power and reliability. We used data from UK Biobank (Allen et al. 2012), a biomedical study based in the United Kingdom. A subset of the full sample of 500,000 participants has contributed neuroimaging data (Miller et al. 2016); a portion of these data have been released for analysis while collection is ongoing, and this subset is analyzed here. We tested male–female differences (in mean and variance) in overall and subcortical brain volumes, mapped the magnitude of sex differences across the cortex with multiple measures (volume, surface area, and cortical thickness), and also examined sex differences in white matter microstructure derived from DT-MRI and NODDI. We tested the extent to which these differences were regionally-specific or brain-general, by adjusting them for the total brain size (or other relevant overall measurement; for instance, adjusting volume differences for total brain volume and cortical thickness differences for mean cortical thickness), and examining whether the differences found in the raw analyses were still present. We tested the extent to which these structural differences (in broad, regional, and white matter measures) mediated sex variation in scores on two cognitive tests, one tapping a mixture of fluid and crystallized reasoning skills (skills previously found to be linked to brain volumes; Pietschnig et al. 2015) and one testing processing speed (previously found to be linked to white matter microstructural differences; see Penke et al. 2012). At the functional level, we also examined large-scale organization of functional networks in the brain using resting-state fMRI functional connectivity data and data-driven network-based analyses.

## Materials and Methods

### Participants

UK Biobank (http://www.ukbiobank.ac.uk/) is a large, population-based biomedical study comprising around 500,000 participants recruited from across Great Britain (England, Scotland, and Wales) between 2006 and 2014 (Allen et al. 2012; Collins 2012; Miller et al., 2016). After an initial visit for the gathering of medical and other information, a subset of these participants began attending for head MRI scanning. MRI data from 5216 participants were available for the present study (mean age = 61.72 years, SD = 7.51, range = 44.23–77.12), collected at an average of around 4 years after the initial visit, and completed on an MRI scanner in Manchester, UK (that is, all data in this analysis were collected on the same scanner; see below for scanner details). There were 2750 females (mean age = 61.12 years, SD = 7.42, range = 44.64–77.12) and 2466 males (mean age = 62.39 years, SD = 7.56, range = 44.23–76.99). Further details regarding the demographics and representativeness of the sample are reported in the Supplemental Materials.

UK Biobank received ethical approval from the Research Ethics Committee (reference 11/NW/0382). The present analyses were conducted as part of UK Biobank application 10 279. All participants provided informed consent to participate. Further information on the consent procedure can be found under the “Resources” tab at the following URL: http://biobank.ctsu.ox.ac.uk/crystal/field.cgi?id = 200.

### Brain Image Acquisition and Processing

MRI data for all participants were acquired on a single Siemens Skyra 3 T scanner, according to previously-reported procedures (Miller et al. 2016; Online Documentation: http://biobank.ctsu.ox.ac.uk/crystal/refer.cgi?id=2367; http://biobank.ctsu.ox.ac.uk/crystal/refer.cgi?id=1977). Briefly, the acquired 3D MPRAGE T1-weighted volumes were preprocessed and analyzed using FSL tools (http://www.fmrib.ox.ac.uk/fsl) by the UK Biobank brain imaging team. This included a raw, de-faced T1-weighted volume, a reduced field-of-view (FoV) T1-weighted volume, and further processing, which included skull stripping, bias field correction and gross tissue segmentation using FNIRT (Andersson et al. 2001, 2007a, 2007b) and FAST (Zhang et al. 2001), yielding cerebrospinal fluid (CSF), grey and white matter volumes. Where large, common artefacts, such as head movement, were identified during scanning, image acquisition was re-started. However, visual quality control was not systematically undertaken by the UK Biobank team; this would be unfeasible due to the very large sample size (Alfaro-Almagro et al. 2018). Subcortical segmentation was also conducted by the UK Biobank imaging team using FIRST (Patenaude et al. 2011) to provide the volumes of 15 structures (atlas in Fig. S1). These data are made available to researchers registered with UK Biobank as a downloadable dataset of Imaging Derived Phenotypes (IDPs). No significant changes were made to scanner hardware or software during the period of MRI data acquisition; full details on protocol phases and relevant upgrades are available at the following URL: http://biobank.ctsu.ox.ac.uk/crystal/docs/brain_mri.pdf.

#### Subregional Analyses

In addition, we used the FoV-reduced T1-weighted volumes from the first release of UK Biobank MRI data to reconstruct and segment the cortical mantle using default parameters in FreeSurfer v5.3 (http://surfer.nmr.mgh.harvard.edu/; Fischl and Dale 2000; Fischl et al. 2004; Ségonne et al. 2007), according to the Desikan–Killiany atlas (Desikan et al. 2006). Visual checking of each segmentation (including tissue identification and boundary positioning errors) was performed at the University of Edinburgh according to the pipeline suggested by the ENIGMA consortium (https://drive.google.com/file/d/0Bw8Acd03pdRSU1pNR05kdEVWeXM/view) and implemented in our previous work (e.g., Cox et al. 2018); we removed severe failures (including global tissue identification errors, for which cases were removed listwise), and also regional parcellation errors (such as minor skull-stripping or boundary-positioning errors, for which ROIs were removed casewise). The volume, thickness, and surface area of all 68 cortical regions of interest (see atlas in Fig. S3) were extracted for 3875 participants. The magnitudes of sex differences across the cortical surface were visualized using the freely-available Liewald-Cox Heatmapper tool (http://www.ccace.ed.ac.uk/research/resources/software-resources/software). We also registered the vertices of each participants’ cortical model to the FreeSurfer average pial surface, smoothed at 20 mm full width half maximum. Vertex-wise regression analyses were then conducted across each aligned cortical vertex for volume, surface area, and thickness using the SurfStat MATLAB toolbox (http://www.math.mcgill.ca/keith/surfstat) for Matrix Laboratory R2014a (The MathWorks Inc., Natick, MA).

#### White Matter Microstructure

MRI (dMRI) acquisitions are openly available from the UK Biobank website in the form of a Protocol (http://biobank.ctsu.ox.ac.uk/crystal/refer.cgi?id=2367), Brain Imaging Documentation (http://biobank.ctsu.ox.ac.uk/crystal/refer.cgi?id = 1977), and in Miller et al. (2016). Following gradient distortion correction, and further correction for head movement and eddy currents, BEDPOSTx was used to model within-voxel multi-fiber tract orientation, followed by probabilistic tractography (with crossing fiber modeling) using PROBTRACKx (Behrens et al. 2003, 2007; Jbabdi et al. 2012). The AutoPtx plugin for FSL (de Groot et al. 2013) was used to map 27 major white matter tracts from which tract-average fractional anisotropy was derived. On the basis of the factor analyses described by Cox et al. (2016), we selected 22 of the white matter tracts for inclusion in the present study. Neurite orientation dispersion and density imaging (NODDI) modeling was conducted using the AMICO tool (https://github.com/daducci/AMICO; Daducci et al. 2015), and the resultant orientation dispersion (OD) maps were registered with the AutoPtx tract masks to yield an average OD value per tract. These measures were also derived by the UK Biobank imaging team and were available as IDPs. An atlas of the selected white matter tracts is provided in Figure S4.

Note that the mean sex differences in the white matter microstructural parameters studied here were already reported by Cox et al. (2016). Here, we add the analyses of variance differences, and the mediation models with diffusion properties as the mediator of the sex difference in cognitive abilities (see below).

#### Resting-state fMRI (rsfMRI)

To analyze resting-state connectivity, we used bulk data from network matrices generated by UK Biobank. As described in the Online Methods section of Miller et al. (2016), participants lay in the scanner and were instructed to “keep their eyes fixated on a crosshair, relax, and “think of nothing in particular””. Data preprocessing, group-Independent Components Analysis (ICA) parcellation, and connectivity estimation were carried out by UK Biobank using FSL packages (http://biobank.ctsu.ox.ac.uk/crystal/refer.cgi?id=1977). The following preprocessing procedures were applied: motion correction using MCFLIRT (Jenkinson et al. 2002), grand-mean intensity normalization using a single multiplicative factor, high-pass temporal filtering with a Gaussian-weighted least-squares straight line fitting (sigma was set as 50.0 s), EPI unwarping using a field map scanned before data collection, gradient distortion correction (GDC) unwarping, and removal of structural artefacts using an ICA-based X-noiseifier (Beckmann and Smith 2004). Any gross preprocessing failure was visually checked and eliminated (Miller et al. 2016). Group-ICA parcellation was conducted on 4162 participants. The preprocessed EPI images were fed into the MELODIC tool in FSL to generate 100 distinct ICA components (45 were removed as noise after visual quality-control, leaving 55 components for analysis). The spatial maps for the components are available at the following URL: http://www.fmrib.ox.ac.uk/datasets/ukbiobank/index.html. Details of preprocessing steps can be found in pages 12, 15 and 16 of Brain Imaging Document (version 1.3) from UK Biobank data showcase website: https://biobank.ctsu.ox.ac.uk/crystal/docs/brain_mri.pdf.

Time series data from the 55 components were used for connectivity analysis, with each component as a node. Two 55 × 55 matrices of fully-normalized temporal correlations and partial temporal correlations were derived for each participant. A larger absolute number indicates stronger temporal connectivity, and the valence represents whether the connection is positive or negative. Partial temporal correlation matrices were used for analysis, as they represent direct connections better than full temporal correlations. Estimation of the partial correlation coefficients was conducted using FSLnets package in FSL (https://fsl.fmrib.ox.ac.uk/fsl/fslwiki/FSLNets). To produce a sparser partial correlation matrix, L2 regularization was applied by setting rho as 0.5 in the Ridge Regression “netmats” option. A description of the settings for the estimations is available at the following URL: http://biobank.ctsu.ox.ac.uk/crystal/refer.cgi?id=9028. To better illustrate the group-average network matrix, the nodes were clustered into 5 categories based on the full-correlation matrices (Miller et al. 2016). The group-average network matrix is shown in Figure S13.

Before analysis of sex differences, we multiplied the strength of each connection by the sign of its group-mean (Smith et al. 2015). For example, where the time series data from two ICA components were positively correlated, but the valence of the connection at the level of the group was negative, the valence for that individual was determined to be negative; that is, individual valences were determined by the valence of that connection at the level of the group. In this way, the valence of the majority of participants’ connections for each node were positive, allowing us to investigate the degree to which temporal connectivity differed by sex without combining positive and negative effects and losing information on the absolute magnitude. We then tested the association of sex with the strength of connections, using the *glm* function in R. As in the other analyses, age and ethnicity were controlled by using them as covariates. Any participant without age or ethnicity information was excluded. 4004 participants were therefore included in this part of the analysis (mean age = 61.63, SD = 7.56; 47.65% male). To assess the importance of the nodes, we generated the weighted degree for a node by calculating the mean strength of its connections with all 54 other nodes. Full results for connection strength (partial and full correlations) and for weighted degree are provided as 3 separate tabs in Table S14. In that table, Cohen’s *d*-values are provided as standardized effect sizes of the sex difference in the strength of connectivity: as for the other analyses, a negative effect size means the strength of the connection was higher in males, and a positive effect size means it was higher in females.

### Cognitive Testing

Cognitive testing took place at the same visit as the MRI scan. Two tests were analyzed here: “fluid intelligence” (henceforth called “verbal-numerical reasoning”), and reaction time. These are described in detail in the Supplemental Materials.

### Statistical Analysis

This section provides a summary of the statistical analyses we ran; a fuller technical description is provided in the Supplemental Materials.

We first adjusted all variables for age and ethnicity (both of which may have been associated with differences in brain measures; Cox et al. 2016; Isamah et al. 2010; Tang et al. 2010). In some analyses, as described below, we adjusted for total brain volume and height. The adjustment techniques are described in the Supplemental Materials.

Welch’s *t*-test was used for the mean comparisons, and a variance ratio test (*F*-test) was used to assess differences in the variance between the sexes. To calculate the associated Cohen’s *d*-value for each *t*-test, we multiplied the *t*-value by 2 and divided it by the square root of the degrees of freedom. The difference between correlations for each sex was calculated using Fisher’s *r*-to-*z* transformation and a *z*-test (using the *r.test* function in the *psych* package for R; Revelle 2016). **P**-values were adjusted, within each analysis and within each brain measure, with the False Discovery Rate correction (Benjamini and Hochberg 1995; for example, the **P**-values for all the sex comparisons on volume were corrected separately from the **P**-values for all the sex comparisons on surface area) using the *P.adjust* function (with the “fdr” correction) for R. We used an alpha level of 0.05 to denote statistical significance. In an additional Bayesian analysis of the mean difference, we used the *BayesFactor* package for R (Morey and Rouder 2015) to compute BF_10_ values from a Bayesian *t*-test (using the *ttestBF* function; see Supplemental Materials).

We used cross-sectional mediation models (in a structural equation modeling framework) to test whether the brain variables (total brain volume, grey matter volume, white matter volume, total surface area, mean cortical thickness, general fractional anisotropy, and general orientation dispersion—the latter two estimated as latent variables—each in separate models, as well as specific brain regions) were significant mediators of the relation between sex and cognitive ability (either verbal-numerical reasoning score or reaction time, in separate models). We also ran multiple-mediator models that used individual brain subregions as mediators of the sex-cognitive relation, instead of overall measures. All methods for running the mediation analyses, along with the equation used to calculate a “percentage of mediation” for each brain variable, are described in the Supplemental Materials.

## Results

### Sex Differences in Overall and Subcortical Brain Volumes

**Figure 1.**
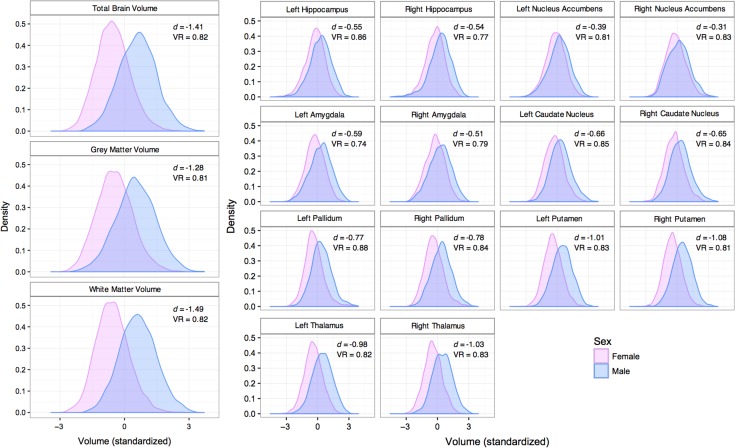
Density plots of sex differences in overall brain volumes (left section) and subcortical structures (right section). *d* = Cohen’s *d* (mean difference); VR = Variance Ratio (variance difference). All mean differences were statistically significant at **P** < 3.0 × 10^−25^, all variance differences were significant at **P** < .003, after correction for multiple comparisons (see Table 1).

The subcortical structures examined were the hippocampus, the nucleus accumbens, the amygdala, the caudate nucleus, the dorsal pallidum, the putamen, and the thalamus (Fig. S1). Raw volumetric sex differences are illustrated in Figure 1. The male distributions were further to the right, indicating higher means, and wider, indicating greater variance. This was confirmed by computing shift functions (Rousselet et al. 2017) for each overall and subcortical brain structure, illustrated in Figure S2a for the raw values and Figure S2b for the values corrected for total brain volume (TBV). There was a substantial degree of overlap between the sexes on all measures.

We first tested for mean sex differences in overall cortical and subcortical brain volumes, adjusting each measure for age and ethnicity (analyses adjusted for TBV were performed separately and are described below). We examined differences in total as well as grey and white matter volumes separately. Differences are shown in Table 1. We observed statistically significant sex differences (adjusted for multiple comparisons using the False Discovery Rate correction), all showing larger volume in males. Note that, in what follows, negative effect sizes indicate higher values for males, and positive effect sizes indicate higher values for females. The effect sizes ranged from small to large; for example, Cohen’s *d* = −0.39 and −0.31 for the left and right nucleus accumbens volume, respectively; −1.41, −1.28, and −1.49 for total, grey matter, and white matter volumes respectively. The average difference for the 14 subcortical volumes was *d* = −0.70. A set of Bayesian *t*-tests (see Supplemental Materials and Table 1) confirmed that the mean sex differences were very large, with extremely strong evidence in favor of the hypothesis that males differed from females on every overall and subcortical volume. We also tested for age-by-sex interactions, assessing whether brain measures were more strongly associated with age in males or females. This was not the case for the overall measures (adjusted **P**-values > 0.8). However, all of the subcortical measures except the amygdala and the caudate showed significant interactions, indicating that the age association was stronger (and the implied age trend steeper) for males. Note that the reported effect sizes come from *t*-tests on variables adjusted for age and sex, but not their interaction.

**Table 1.** Descriptive statistics with mean and variance comparisons for overall volumes, subcortical volumes, and cognitive tests

Measure type	Measure	Female (*n* = 2750)	Male (*n* = 2466)	Mean difference test				Variance Ratio test	
		M (SD)	M (SD)	*t*	*P*	*d*	BF_10_	VR	*P*
Overall volumes (cm^3^)	Total brain volume	1115.76 (89.68)	1233.58 (98.31)	–48.91	~0.00	–1.41	9.57 × 10^426^	0.82	6.46 × 10^–06^
	Grey matter volume	597.02 (47.78)	643.45 (52.08)	–38.97	1.75 × 10^–287^	–1.28	1.62 × 10^289^	0.81	3.60 × 10^–06^
	White matter volume	518.85 (47.89)	589.59 (52.69)	–51.53	~0.00	–1.49	1.47 × 10^465^	0.82	7.31 × 10^–06^
Subcortical volumes (cm^3^)	Left hippocampus^a^	3.73 (0.42)	3.94 (0.46)	–18.91	2.69 × 10^–76^	–0.55	1.09 × 10^74^	0.86	3.83 × 10^–04^
	Right hippocampus^a^	3.82 (0.42)	4.04 (0.48)	–18.43	1.16 × 10^–72^	–0.54	7.97 × 10^70^	0.77	1.16 × 10^–09^
	Left accumbens^a^	0.49 (0.11)	0.53 (0.12)	–13.42	5.19 × 10^–39†^	–0.39	2.13 × 10^36^	0.81	2.95 × 10^–06^
	Right accumbens^a^	0.40 (0.10)	0.42 (0.11)	–10.64	3.82 × 10^–26†^	–0.31	1.04 × 10^23^	0.83	4.46 × 10^–05^
	Left amygdala	1.21 (0.22)	1.35 (0.25)	–20.04	5.23 × 10^–85†^	–0.59	4.73 × 10^83^	0.74	5.89 × 10^–12^
	Right amygdala	1.18 (0.24)	1.31 (0.27)	–17.55	2.16 × 10^–66†^	–0.51	1.60 × 10^64^	0.79	1.54 × 10^–07^
	Left caudate	3.28 (0.38)	3.54 (0.41)	–23.00	3.04 × 10^–110^	–0.66	2.70 × 10^108^	0.85	2.38 × 10^–04^
	Right caudate	3.45 (0.40)	3.72 (0.44)	–22.67	2.37 × 10^–107^	–0.65	4.08 × 10^105^	0.84	4.46 × 10^–05^
	Left pallidum^a^	1.69 (0.21)	1.85 (0.22)	–26.64	4.87 × 10^–145†^	–0.77	2.19 × 10^143^	0.88	.002
	Right pallidum^a^	1.74 (0.20)	1.89 (0.22)	–26.96	3.82 × 10^–148†^	–0.78	8.59 × 10^146^	0.84	1.03 × 10^–04^
	Left putamen^a^	4.61 (0.50)	5.07 (0.56)	–34.72	1.73 × 10^–234†^	–1.01	1.29 × 10^235^	0.83	1.46 × 10^–05^
	Right putamen^a^	4.64 (0.49)	5.13 (0.55)	–37.13	4.76 × 10^–264†^	–1.08	3.02 × 10^265^	0.81	1.98 × 10^–06^
	Left thalamus^a^	7.54 (0.64)	8.11 (0.72)	–33.73	7.76 × 10^–223^	–0.98	1.50 × 10^223^	0.82	1.34 × 10^–05^
	Right thalamus^a^	7.34 (0.62)	7.92 (0.69)	–35.76	2.42 × 10^–247^	–1.03	6.62 × 10^247^	0.83	4.46 × 10^–05^
Cognitive tests	Verbal-numerical reasoning (max. score 13)	6.80 (2.10)	7.14 (2.13)	–6.21	5.77 × 10^–10^	–0.18	6.94 × 10^6^	0.97	.451
	Reaction time (ms)	590.37 (98.04)	574.71 (100.71)	–7.63	2.71 × 10^–14^	–0.21	1.30 × 10^11^	0.92	.033

*Note*: Means and SDs are shown prior to adjustment for age and ethnicity; statistical tests are performed after this adjustment. Reaction Time is shown here in raw millisecond units, but was reverse-scored for analysis so that higher scores indicated better performance. Negative *t*- and *d*-values mean higher male means. VR = Variance ratio (values < 1 indicate greater male variance). **P**-values for brain variables corrected for multiple comparisons using the False Discovery Rate correction. BF_10_ = Bayes Factor indicating the probability of the alternative hypothesis (that there is a sex difference) compared to the null hypothesis (that there is no sex difference). ^a^significant age-by-sex interaction. ^†^sex difference in subcortical region still significant after adjustment for total brain volume (see Table S1).

We tested whether sex differences in the subcortical measures were accounted for by the substantial difference in total brain volume. We regressed each subcortical variable on total brain volume, testing these adjusted residuals for sex differences. After this adjustment, there were no longer statistically significant differences in the hippocampus, caudate nucleus, or thalamus (all **P**_adj_-values > 0.60, absolute *d*-values < 0.03; Table S1). There remained differences in each of the other measures, albeit with attenuated effect sizes (average *d* for significant differences after adjustment = 0.17). Females had greater nucleus accumbens volume after adjustment for total brain volume (*d* = .08, **P**_adj_ = .07 for left accumbens; *d* = 0.10, **P**_adj_ = 0.003 for right). Overall, the majority of the sex differences in specific subcortical structures appeared to be linked to the difference in total brain size (average pre-adjustment attenuation of *d*-values for subcortical structures = 85.0%). We also ran analyses adjusting for height, since overall body size may have influenced these differences (as expected, males were substantially taller on average: *d* = −2.15). This attenuated all of the *d*-values (average attenuation across global and subcortical measures = 71.3%), but males still showed significantly larger volumes for all subcortical regions except the nucleus accumbens (Table S1). For example, post-adjustment *d*-values were −0.42 for total brain volume, −0.31 for grey matter volume, and −0.47 for white matter volume. The Bayesian analyses, also shown in Table S1, again confirmed these results: for instance, showing more evidence for the null hypothesis of no difference for the bilateral nucleus accumbens after adjustment for height, but still showing substantially more evidence for the hypothesis of a difference for all other regions.

As shown in Table 1, there were statistically significant variance differences in all overall cortical and subcortical brain volumes, with males showing greater variance; the average variance ratio for overall volumes and subcortical volumes was 0.82 (variance ratios <1.00 indicate greater male variance). After adjusting for total brain volume or height, the variance differences reported in Table 1 remained relatively unchanged (see Table S1).

### Sex Differences in Subregional Brain Volume, Surface Area, and Cortical Thickness

**Figure 2.**
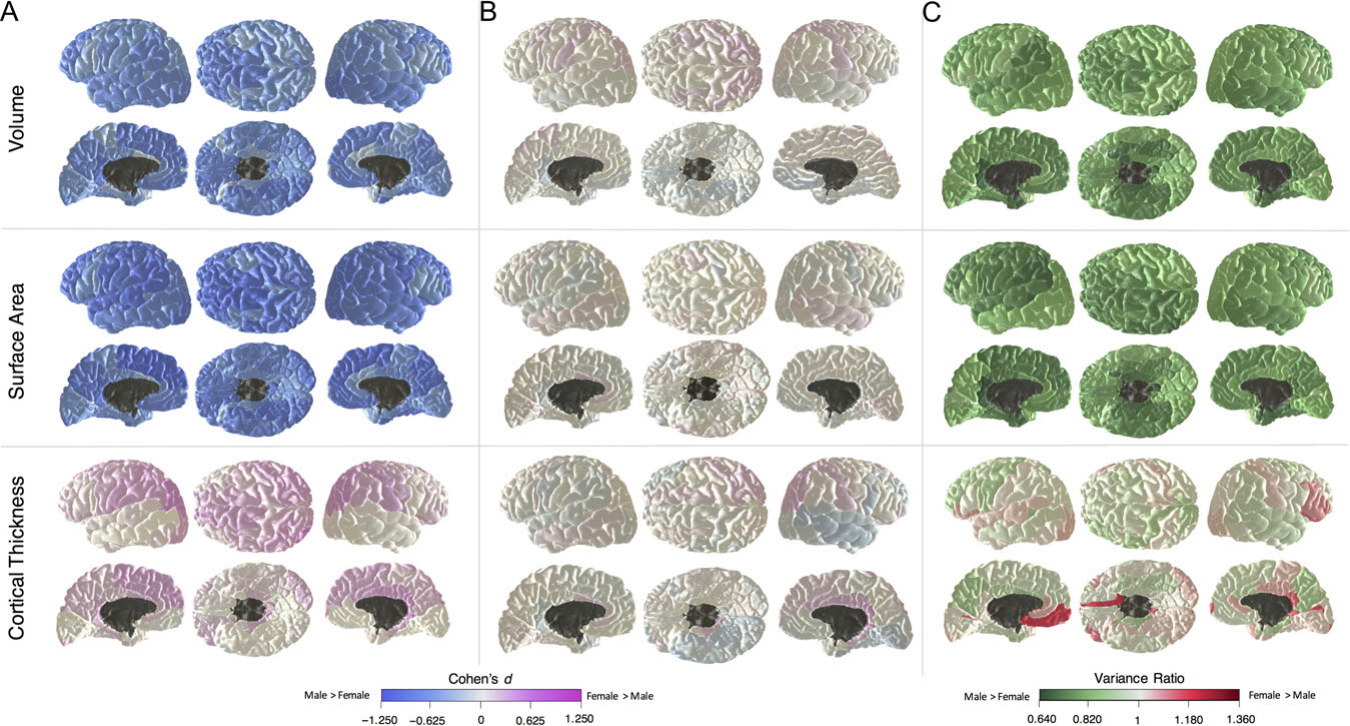
Sex differences across the subregions in volume, surface area, and cortical thickness. Shown are (*A*) mean differences, (*B*) mean differences adjusted for total brain volume, total surface area, and mean cortical thickness (respectively by column); and (*C*) variance differences. Adjusted variance differences were near-identical to those shown in (*C*); see Figure S5. See Figure S3 for subregional atlas.

Using FreeSurfer to parcellate cortical regions according to the Desikan–Killiany neuroanatomical atlas (Desikan et al. 2006; S3), we tested for sex differences in (raw) volume, surface area, and cortical thickness across 68 cortical subregions. As with the analyses above, we adjusted all subregions for age and ethnicity; **P**-values were also adjusted within each measure type using the False Discovery Rate correction. The results are illustrated in Figure 2*A* (see also Table S2 for means, standard deviations, and difference tests for volume, surface area, and cortical thickness across all cortical regions).

Males showed larger brain volume across all cortical subregions. The sex difference was statistically significant in every subregion, ranging in size from small (*d* = −0.24 in the right temporal pole) to large (*d* = −1.03 in the right insula). The mean *d*-value across all subregions was −0.67 (**P**_adj_-values < 9.00 × 10^−13^). Even larger differences, all favouring males, were observed for surface area; these ranged from moderate (*d* = −0.43 in the left caudal anterior cingulate) to large (*d* = −1.20 in the left superior frontal region). The mean *d*-value across all subregions was −0.83 (all **P**_adj_-values < 2.00 × 10^−36^).

Cortical thickness displayed a different pattern. Unlike volume and surface area, females had thicker cortex across almost the entire brain. The only area where males showed a statistically significantly thicker cortex was the right (but not left) insula, and the difference was small (*d* = 0.14). In all other areas, there was either no significant thickness difference (20/68 areas), or a statistically significant difference favouring females. The mean *d*-value in the 47 areas that reached statistical significance after multiple-comparisons correction was 0.22, ranging from *d* = 0.07 in the right rostral middle frontal region to *d* = 0.45 in the left inferior parietal region. Overall, higher female cortical thickness was generally not found in the temporal lobe (except the parahippocampal gyrus) or in the medial orbitofrontal regions. In some regions there appeared to be converse differences: in the motor and somatosensory regions in the parietal lobe, the frontal pole, and the parahippocampal gyrus, females showed relatively higher thickness but males showed relatively higher volume and surface area. In the superior temporal lobe and orbitofrontal regions, males showed relatively higher volume and surface area, but there was no particular sex difference in thickness. Once more, all of the subregional analyses were confirmed using the alternative Bayes Factor analyses.

We also tested age-by-sex interactions for each of the 3 variables (volume, surface area, and cortical thickness; Table S2). After multiple-comparisons correction, only two interactions were significant: the left and right superior frontal regions showed significantly stronger volume relations with age in males. That is, males may have had steeper volume decline in this region bilaterally with age. There were no statistically significant age-by-sex interactions for surface area or cortical thickness.

We next adjusted the subregional volume, surface area, and cortical thickness measures for total brain volume, total surface area, or mean cortical thickness, respectively. As shown in Figure 2*B* (and Table S3, which also shows the surface area and cortical thickness measures adjusted for total volume, since this is a common procedure in neuroimaging analyses), 11 regions were still significantly larger in volume for males (mean *d* = −0.14; the largest being the left isthmus cingulate, *d* = −0.22). There were also 13 regions where females now had a significantly larger volume (mean *d* = 0.15; the largest difference being in the right superior parietal, *d* = 0.21). The majority of regions (44/68) no longer showed significant volume differences (all **P**_adj_-values > .34). For surface area, males were significantly larger in 18/68 areas after total surface area adjustment (mean *d* = −0.12, the largest difference in the right pars orbitalis; *d* = −0.16), and females were larger in 9 (mean *d* = 0.11; the largest being the left caudal anterior cingulate, *d* = 0.18). The remaining 41 surface areas showed no significant difference (all **P**_adj_-values > .05) For cortical thickness, after correction for mean thickness there were still significant differences favouring females in 24/68 regions (mean *d* = 0.17; the largest in the left inferior parietal, *d* = 0.32), but also 25 regions with differences favouring males (mean *d* = −0.16, the largest in the right insula, *d* = −0.34). Next, we adjusted the cortical subregional measures for height (Table S4). For volume, all of the comparisons were still significant, but with reduced effect sizes (−0.33 < *d <* −0.07, mean *d* = −0.19, all **P**_adj_-values < .05); this was the same for surface area (−0.35 < *d* < −0.10, mean *d* = −0.25, all **P**_adj_-values < .002). For thickness, there were 34/68 regions that were still significantly thicker in females (mean *d* = 0.12, the largest in the left inferior parietal, *d* = 0.19), and one thicker in males (the left entorhinal cortex, *d* = −0.08).

Variance differences across the 3 structural measures are illustrated in Figure 2*C*. For volume and surface area, males showed significantly greater variance than females across almost all brain regions. The volume variance ratio was significant in 64/68 regions, ranging from 0.88 in the right temporal pole to 0.67 in the left isthmus cingulate, with all **P**_adj_-values < .031 after correction. The surface area variance ratio was significant in 66/68 regions, ranging from 0.88 in the left pars orbitalis to 0.65 in the left isthmus cingulate, all **P**_adj_-values < .018 after correction. For cortical thickness (Fig. 2*C*), there were no significant variance differences in any region (all **P**_adj_-values > .14) except one: females showed significantly greater variance in the thickness of the left medial orbitofrontal cortex (VR = 1.19, **P**_adj_ = .01). As can be observed from Figure S5 (and Table S3), controlling for the respective overall measures made only a negligible difference to the pattern of variance ratios reported above.

We tested whether the regions showing larger mean differences were also those with larger variance differences, by correlating the vector of *d*-values with the vector of VRs for each brain measure. As shown in Figures S6 and S7, there was some correspondence between mean and variance: in the unadjusted analysis, mean and variance were correlated at *r* = .51 for volume, but there were smaller correlations for surface area and thickness (*r*-values = .25 and −.06, respectively). Adjusted for TBV, all 3 correlations were relatively weak (*r*-values = .22, .03, and −.25 for the 3 brain measures respectively).

To verify whether the pattern of results across the cortical mantle was agnostic to the gyral boundaries of the Desikan–Killiany atlas, we conducted a supplemental analysis, testing sex differences using a vertex-wise approach, the results of which are shown in Figures S8 (for mean differences) and S9 (for variance differences). This precisely replicated the subregional atlas-based results.

### Sex Differences in White Matter Microstructure

**Figure 3.**
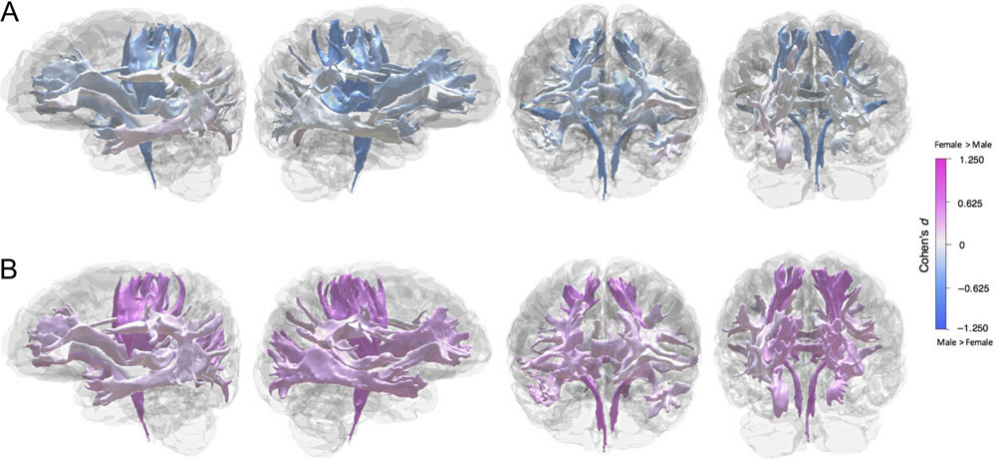
Mean sex differences in white matter microstructural measures (*A*) fractional anisotropy and (*B*) orientation dispersion across 22 white matter tracts. For both measures, numerically the largest effect was found in the right cortico-spinal tract. See Figure S4 for tract atlas.

We tested sex differences in 22 white matter tracts. We focused on two white matter microstructural properties that had previously been shown to demonstrate differences between males and females in the initial release of UK Biobank imaging data (Cox et al. 2016). The first was fractional anisotropy (FA), an index of the directionality of water diffusion through the white matter. The second was orientation dispersion (OD), a NODDI measure of white matter tract complexity. For FA, there were generally higher values in males, particularly in the cortico-spinal tract (*d* = −0.54) and the acoustic radiation (*d* = −0.51). The average difference across tracts was *d* = −0.19. OD was higher in all tracts for females (average *d* = 0.30). These mean differences are shown in Figure 3, and fully reported in Tables S5 and S6.

Variance differences are illustrated in Figure S10 (see also Tables S5 and S6). Generally, there was greater male variance in FA (average VR = 0.92); however, there was substantially greater female variance in the cortico-spinal tract in particular (VR = 1.17, **P** = .0003). For OD, the only tract that showed a significant variance difference following FDR correction was the left superior thalamic radiation, where males showed greater variance (VR = 0.79).

Adjusting the microstructural measures attenuated the size of the mean sex differences in FA (in one case producing a significantly higher score in females that was not present in the raw analysis, for the left inferior fronto-occipital fasciculus; Table S5). For OD, this adjustment made very few changes to the mean differences (Table S6). The TBV adjustment left the variance differences broadly unchanged—in some cases with a small increase in the size of the sex difference—for both FA and OD. This attenuation in the size of the mean sex differences in FA, but not OD, after adjustment for TBV may be a result of FA’s sensitivity to “partial volume” effects.

### Relation of Neurostructural Differences to Cognitive Differences

We linked the structural brain differences to scores on two cognitive tests taken at the time of the imaging visit: verbal-numerical reasoning and reaction time (see Method). Descriptive statistics for the cognitive tests are shown in Table 1. Note that we coded both tests (reflecting the reaction time variable) so that higher scores indicated better performance. The test scores correlated positively, but weakly (*r* = .12). Males had a slightly higher mean score than females on verbal-numerical reasoning (*d* = −0.18) and slightly faster mean reaction time (*d* = −0.22); there was no significant variance difference for verbal-numerical reasoning (VR = 0.97, **P** = .45), though males had marginally more variance in reaction time (VR = 0.92, **P** = .03).

As a first step toward the mediation analyses, we correlated performance on the two cognitive tests with the overall brain measures in the full sample (Table S7), and in two randomly-selected sample halves separately (Table S8). The sample was split in this way to avoid overfitting and assess the replicability of the results. We then ran the same correlations across all the raw brain subregions, for volume, surface area, and cortical thickness (Table S9). These correlations were generally small, with all brain-cognitive *r*-values <.20. We compared the size of the correlations across the sexes; after multiple comparisons correction, there were no significant sex differences in these correlations. Thus, there was no evidence in the present analysis for sex differences in how regional brain structure related to the two measured cognitive skills.

Next, we tested the extent to which the mean cognitive differences were mediated by any of the overall brain measures (total, grey, and white matter volumes, total surface area, mean cortical thickness, or general factors of FA or OD). We ran a separate model, illustrated in Figure S11, for each brain measure. Results are displayed in Tables S10 and S11 for verbal-numerical reasoning and reaction time, respectively. For verbal-numerical reasoning, the sex difference in test scores was mediated substantially by brain volume measures and by surface area (all mediation percentages >82%). Cortical thickness showed far smaller mediation percentages (7.1% and 5.4% in the two sample halves, respectively). For reaction time, total brain and white matter volumes had mediation percentages >27%, but the other measures all produced smaller percentages (<15.3%), particularly mean cortical thickness (mediating <3% of the variance).

**Figure 4.**
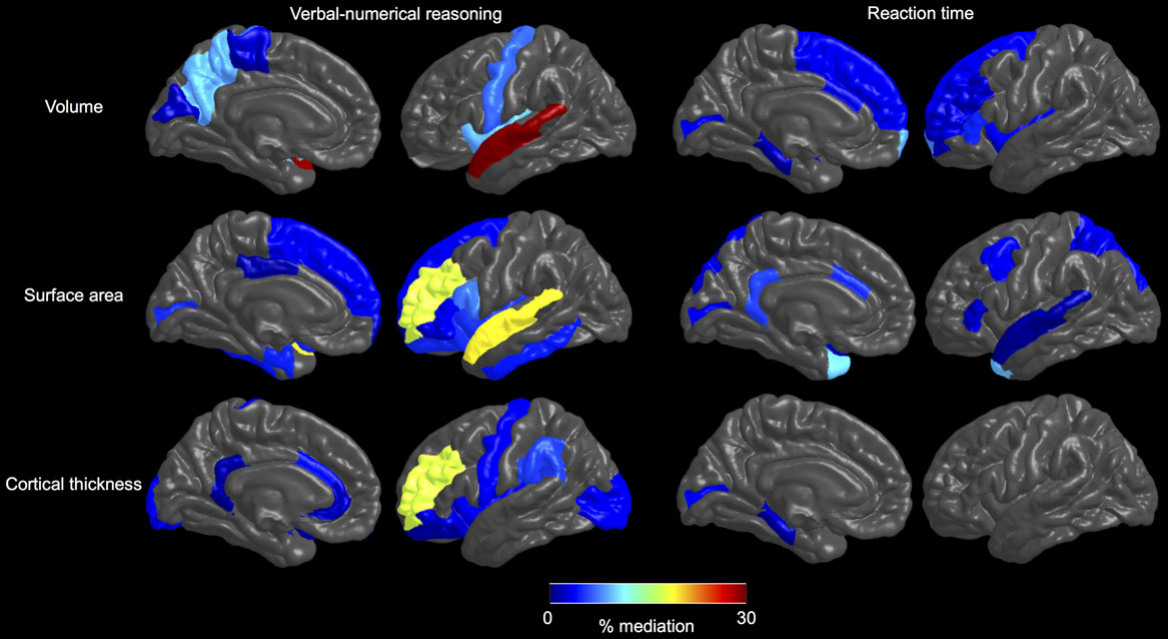
Percentage of the sex-cognitive relation mediated by each of the brain regions selected in a LASSO model to be linked to either verbal-numerical reasoning (left column) or reaction time (right column). Results for volume, surface area, and cortical thickness are shown in each row. Regions were averaged across the hemispheres; thus only a medial and lateral view for each measure and each cognitive test is shown.

Finally, we tested which (raw) brain subregions were most important in explaining the mediation of the sex-cognitive relation, by running mediation models that included multiple individual regions as mediators. These variables were selected for their association with the cognitive ability in question (again, either verbal-numerical reasoning or reaction time) using LASSO regression models (see Method for details). The percentage of mediation for each selected region is illustrated in Figure 4 (see Figure S12 and Table S12 for full results). For verbal-numerical reasoning, the volume and surface area of the superior temporal region mediated the largest amounts of variance (29.1% and 18.4% in their respective models), with other relatively substantial contributions coming from the precuneus and insula for volume, and the pars opercularis and rostral middle frontal regions for surface area. For the cortical thickness predictors, and for the outcome of reaction time, as expected on the basis of the overall mediation results reported above, few of the regions showed substantial mediation (there was some mediation by the volume of frontal regions; at most 7.3% by the frontal pole).

### Sex Differences in Resting-state Functional Connectivity

**Figure 5.**
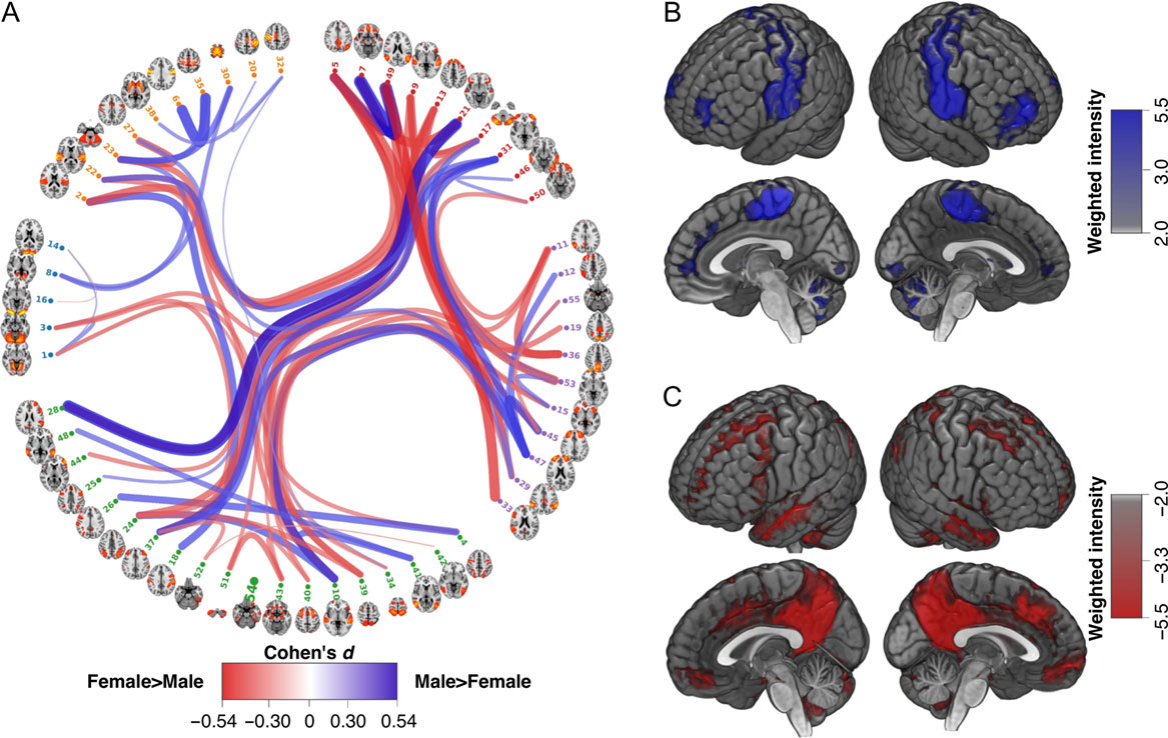
Results for resting-state *f*MRI connectivity and weighted degree of nodes. (*A*) Spatial maps for individual connections. Colors and line thickness represent the effect sizes of sex on the strength of connections (red = stronger in females; blue = stronger in males; darker/thicker = larger effect size). Only effect sizes (Cohen’s *d*) larger than ±0.2 are shown. Nodes were clustered into 5 categories using FSLnets based on their group-mean full-correlation matrix (yellow/orange: sensorimotor network; red: default mode network; purple: salience network and executive control network; green: dorsal attention network; blue: visual network). (*B*) and (*C*) Weighted degrees of nodes with higher values in males and females, respectively. The spatial maps of significant group-ICA nodes were multiplied by the effect size of the sex correlation. In order to show the regions with the largest associations with sex, only regions that had intensity over 50% of the whole-brain peak value are presented. See Table S14 for values for each connection and for each node’s weighed degree.

For our final set of analyses, we examined sex differences in resting-state functional MRI (rsfMRI) responses within a number of functional networks. The connections between each pair of functional networks were estimated and then transformed into measures of strength (see Method). We found that 54.7% (811 of 1485) of network connections showed a statistically significant sex difference (absolute *β*-values = 0.071–0.447 for females; 0.071–0.519 for males). A map showing the strengths of the connections between the 55 network nodes, and whether the difference was stronger in males (blue) or females (red) is provided in Figure 5*A* (see also Table S13). The strength of connectivity between sensorimotor, visual, and rostral lateral prefrontal areas was absolutely higher in males than females (see the cluster of brain regions with orange numerals in Fig. 5*A*), whereas the strength of connectivity within the default mode network (DMN; cluster of regions with red numerals in Fig. 5*A*) was absolutely higher in females than males.

To further analyze these functional sex differences, we calculated the mean strength of all 54 connections to each individual node, producing a “weighted degree” statistic. Sex differences in weighted degree are shown in Figure 5*B*,*C*. Males showed stronger weighted degree than females in bilateral sensorimotor areas, the visual cortex, and the rostral lateral prefrontal cortex. Females showed stronger weighted degree than males in cortical areas comprising the DMN: the bilateral posterior cingulate cortex/precuneus, the dorsal anterior cingulate cortex, medial prefrontal cortex, temporo-parietal junction, anterior temporal lobe, medial temporal lobe (e.g., hippocampus and surrounding areas), and some cerebellar regions (see Tables S13 and S14).

## Discussion

In a single-scanner sample of over 5000 participants from UK Biobank, we mapped sex differences in brain volume, surface area, cortical thickness, diffusion parameters, and functional connectivity. One main theme of the neurostructural results was that associations with sex were global. Males generally had larger volumes and surface areas, whereas females had thicker cortices. The differences were substantial: in some cases, such as total brain volume, more than a standard deviation. The effect size of *d* = −1.41 for total brain volume (Table 1) translates to 92.1% of males being above the female mean, and an 84.1% chance that a randomly-chosen male will have a larger total brain volume than a randomly-chosen female. (The following online app can be used to calculate further such values: http://rpsychologist.com/d3/cohend/.)

We also found that volume and surface area mediated nearly all of the small sex difference in reasoning ability, but far less of the difference in reaction time. For white matter microstructure, females showed lower directionality (FA) and higher tract complexity (OD); white matter microstructure was a poor mediator of the cognitive sex difference. Resting-state fMRI analyses also revealed a global effect: around 54% of connections showed a sex difference. These differences clustered around specific networks, with stronger connectivity in females in the default mode network and stronger connectivity in males between unimodal sensory and motor cortices as well as high-level cortical areas in the rostral lateral prefrontal cortex. For every brain measure that showed even large sex differences, there was always overlap between males and females (see Fig. 1): even in the case of the large difference in total brain volume noted above, there was 48.1% sample overlap.

The principal strengths of the present study are its sample size (providing sensitivity for the identification of small effects with high statistical power), the wide range of MRI modalities, and the consideration of both mean and variance differences. Given the surfeit of small-*n* studies in neuroscience (Button et al. 2013; Nord et al. 2017), it is of great importance to test hypotheses in large, well-powered samples, especially given that many neural sex differences are modest in size (Joel et al. 2015). Here, we had excellent statistical power to find small effects in brain subregions, providing a robust and detailed analysis. For our subregional analysis, we had a far larger sample size than the most recent meta-analysis (Ruigrok et al. 2014). In contrast to that meta-analysis—which found greater volume for females in areas such as the thalamus, the anterior cingulate gyrus, and the lateral occipital cortex—our study found no brain subregions where females had a larger volume than males. The reason for this may be the more restricted age range of the participants in our study (sex may relate differently to the brain at different ages, as we found for several brain regions in our age-by-sex interaction analyses, and as was found in a previous developmental study of children and adolescents; Gennatas et al. 2017) or, more likely, study size and heterogeneity: the data for section of the meta-analysis on regional volumes came from many separate studies, on separate scanners, generally with small sample sizes (many with *n* < 100), whereas our contrasts were based on one very large, single-scanner study.

The higher male volume in our study appeared largest in some regions involved in emotion and decision-making, such as the bilateral orbitofrontal cortex, the bilateral insula, and the left isthmus of the cingulate gyrus (Craig 2009; MacPherson et al. 2015; Ochsner and Gross 2005; Wager et al. 2008; note that the insula showing the largest sex difference is consistent with a recent large-scale study of children and adolescents (Gennatas et al. 2017)—it appears this region retains its substantial sex difference into later life), but also areas such as the right fusiform gyrus. For surface area, which showed an even larger difference favouring males, the regions that showed the largest effects were broadly areas involved in the hypothesized intelligence-related circuit in the “P-FIT” model (Jung and Haier 2007): for example, the bilateral superior frontal gyri, the bilateral precentral gyri, the left supramarginal gyrus, and the bilateral rostral middle frontal areas. However, some of the regions involved in this theorized circuit were also larger, in terms of thickness, for females. For instance, the bilateral inferior parietal regions were the regions with numerically the largest difference favouring females in cortical thickness. Our finding that raw cortical thickness was greater for females—a finding with a smaller mean effect than the one in the opposite direction for volume or surface area—is consistent with a number of previous, smaller studies (e.g., Luders et al. 2006; Sowell et al. 2007; Lv et al. 2010; van Velsen et al. 2013; though not others: Salat et al. 2004; Reid et al. 2010). Our greater statistical power may have allowed us to find smaller differences in thickness across the cortex. It is interesting to note that a previous study from the animal literature found greater cortical thickness in male mice (Markham et al. 2003); however, this study had a small sample size and by necessity used very different methods from ours: further comparison of sex differences in the brains across different species might reveal more about the cellular mechanisms underlying these differences.

Whereas previous work has found some white matter regions where fractional anisotropy was higher for females (Kanaan et al. 2012; Dunst et al. 2014), we found that males showed higher FA in 18 of the 22 tracts we examined. FA also generally showed greater variance in males. On the other hand, higher orientation dispersion was found for females in all tracts. Unexpectedly, higher OD was found to be related to lower cognitive performance on the two tests examined here. Since OD is a relatively new measure of white matter microstructure (Daducci et al. 2015), further work should aim to clarify its behavioral correlates. The fact that (as described in the Method section) measurement invariance did not hold across the sexes for the latent variables of FA and OD, indicating that the tract-specific measurements may be assessing somewhat different latent variables in each sex, may also be of interest for future researchers examining general-level indicators of white matter microstructure.

The issue of adjusting for overall brain size in analyses of sex differences (Rippon et al. 2014) was addressed in each of our macrostructural analyses. As can be seen comparing Figure 2*A* and *B*, after this adjustment the higher male volume and surface area was substantially reduced, often to non-significance. For those latter brain regions, this implies that the sex difference was general and that the larger volume or surface area was a by-product of the overall larger male brain. However, for some regions, especially for surface area (particularly in areas such as the left isthmus of the cingulate gyrus and the right precentral gyrus), males still showed a significantly higher measurement, indicating specific sex differences in the proportional configuration of the cortex, holding brain size equal. Most interestingly, for some areas (for example the right insula, the right fusiform gyrus, and the left isthmus of the cingulate gyrus), the difference was reversed after adjustment, with females showing significantly larger brain volume.

A recent meta-analysis of sex differences in amygdala volume (Marwha et al. 2016) found that, although males showed larger raw volume, after correction for total brain volume there was no longer an appreciable sex difference. However, in our study the amygdala was significantly, but modestly, larger in males even after adjusting for total brain volume (*d* = 0.18 bilaterally). The heterogeneity in the methods of the studies being meta-analyzed may have led to the divergent conclusion from our single-sample study. With regard to the hippocampus, however, we found results consistent with another recent meta-analysis (Tan et al. 2016): there were no longer significant sex differences after adjustment for total brain volume (this was also the case for the thalamus and caudate). We recommend that future studies perform comparisons both before and after adjusting for total volume (or the relevant overall measure, in the case of measures such as cortical thickness or surface area), since these results pertain to quite different questions: unadjusted, they ask which regions differ in an absolute sense; adjusted, they ask which regions are different relative to the overall size (or other overall measure) of the brain. Both of these may be important questions to ask in different contexts.

One question that could not be addressed using the current data regards the underlying bio-social causes, ultimate or proximate, for the sex differences that we observed. Many variables were collected in UK Biobank that might be linked to the sex differences observed here (and may be proximal causes of them) but our intention in the present study was to characterize, not necessarily explain, these differences: future research should investigate more targeted hypotheses of the causes of the differences. Sex differences in brain structure are observed early in the life course (e.g., Knickmeyer et al. 2014), though this does not imply that the pattern of adult differences we observed is necessarily the same as is found in childhood. The literature on developmental sex differentiation of the brain highlights influences of factors, such as genes on sex chromosomes or sex hormones acting via receptors throughout the developing and adult brain, that were not analyzed in the present study (McCarthy and Arnold 2011; Lombardo et al. 2012; McEwen and Milner 2017). Likewise, understanding the potential neurobiological effects of social influences during development (Dawson et al. 2000) was beyond the scope of our research and our dataset.

Our analysis also focused on sex differences in variance. Here, for the first time in an adult sample, we directly tested sex differences in the variance of several brain measures, finding greater male variance across almost the entire brain for volume, surface area, and white matter fractional anisotropy, but only patchy and inconsistent variance differences for cortical thickness and white matter orientation dispersion. Our correlation of the vector of mean differences with that of variance ratios showed that there was some degree of correspondence between them for volume, but the relation was far smaller for surface area (and was near-zero for cortical thickness). In all cases, this analysis indicated that mean and variance differences appear to be largely independent aspects of sex differences in the brain; we should not expect to see that areas showing the largest difference in mean also show the largest difference in variance, at least to a great extent.

One potential candidate to explain greater male variability across multiple phenotypes is the hypothesized “female-protective” mechanism involving effects of the *X* chromosome (Craig et al. 2009; Johnson et al. 2009; Reinhold and Engqvist 2013), or other protective factors that might “buffer” females from potential deleterious consequences of rare genetic mutations (Jacquemont et al. 2014; Robinson et al. 2013). Such explanations are speculative at present; as studies like UK Biobank release even larger amounts of data on individuals who have both neurostructural and genotype data, researchers will be able to perform well-powered tests of these hypotheses.

In discussing any potential genetic effects on sex differences, analyses should take into account the fact that such effects are likely active at multiple points across the lifespan, representing a continuing, complex set of influences that may interact with environment and experience. This complexity is deepened by phenomena such as genomic imprinting (where the expression profiles of certain genes differs according to whether they were inherited paternally or maternally, which has been hypothesized to contribute to the aforementioned sex-biased nature of some psychiatric disorders; Dulac and Christopher 2013) and the “mother’s curse” (a buildup of male-specific mutations in mitochondrial DNA, which is passed on maternally; Gemmell et al. 2004).

Using the (limited) data on cognitive abilities available in our sample, we tested whether the data were consistent with any consequences of brain structural differences in terms of ability differences. There were only weak correlations between brain variables and the cognitive tests (consistent with previous evidence of these links: Karama et al. 2014; Schnack et al. 2014; Pietschnig et al. 2015), and these associations did not differ by sex (consistent with the brain-volume-cognitive meta-analysis of Pietschnig et al. 2015). Mediation modeling suggested that, for verbal-numerical reasoning, a very large portion (up to 99%) of the modest sex difference was mediated by brain volumetric and surface area measures. Smaller fractions (up to 38%) of the modest link between sex and reaction time could be explained by volume or surface area. Perhaps unexpectedly, given evidence and theory linking white matter microstructure to cognitive processing speed (Penke et al. 2012; Bennett and Madden 2014), white matter microstructural measures only mediated a small proportion of the sex difference in reaction time (this may have been due to weaknesses in this cognitive measure; see below). Cortical thickness had trivial mediating effects compared to volume and surface area: no more than 7.1% of the sex-cognitive relation was mediated by thickness in any analysis. With our multiple-mediator models, we built a map of which brain regions were most relevant in this mediation of the sex-cognitive relation (Fig. 4). Overall, the data were consistent with higher volume and cortical surface area—but not cortical thickness or microstructural characteristics—chiefly in the superior temporal region, but also spread across multiple other regions to a lesser extent, being of particular relevance to sex differences in reasoning (but not reaction time).

An additional hypothesis—one that is not incompatible with the hypothesis that some of the sex differences seen here are a proximate cause of behavioral differences—is that brain structural differences might sometimes be the result of compensatory mechanisms for differences in sex-specific hormones, and might thus act to *reduce* behavioral sex differences that would otherwise have been present (De Vries 2004; McCarthy and Arnold 2011). This perspective may in part explain an apparent paradox in human sex difference research: that the (raw) effect sizes found for brain measures such as volume and surface area are so large, whereas most behavioral sex differences are so small (Hyde 2014). Our descriptive results do not directly speak to any causal mechanisms, but it should be borne in mind that they are compatible with these multiple interpretations.

Sex differences in intrinsic functional connectome organization also revealed results that corroborate and extend prior work. We successfully replicated the results from the 1 000 Functional Connectomes dataset (an entirely separate dataset)—that is, we found female > male connectivity within the default mode network and some evidence for male>female connectivity in sensorimotor and visual cortices (Biswal et al. 2010). The higher female connectivity within circuits like the DMN may be particularly important, given that DMN regions are often considered as an important part of the “social brain” (Kennedy and Adolphs 2012; Mars et al. 2012; Amft et al. 2015). Whether such an effect can help explain higher average female ability in domains like social cognition (Gur et al. 2012), and whether such functional differences can be integrated with differences in the structural connectome (Ingalhalikar et al. 2014), remains to be seen. Finally, recent work has shown that intrinsic functional connectome organization can be parsimoniously described as a small number of connectivity gradients (Margulies et al. 2016). The most prominent connectivity gradient has at one pole the DMN and at the other unimodal sensory and motor cortices. The observed pattern of sex differences in functional connectome organization observed here appears to recapitulate the two main poles of that principal connectivity gradient (Margulies et al. 2016). One potential way of describing the biological significance of these functional sex differences is that mechanisms involved in shaping sex differences (biological, cultural, or developmental) may influence this principal connectivity gradient; the result, which should be explored in future investigations of brain sex differences, may be the multiple network differences found in the present study.

## Limitations

The UK Biobank sample was selective. It covered only one part of the life course (from approximately 45 to 75 years of age), and thus our findings may not apply to younger adults. With ageing may come larger variation in some brain parameters (Cox et al. 2016), meaning that the distributions found here may not fully represent those across the lifespan. Many of the female participants might have been undergoing, or have undergone, menopause; this (or associated Hormone Replacement Therapy) might exert modest effects on the structure of some regions of the brain (Zhang et al. 2016), effects which may themselves change with increasing age. In addition, UK Biobank had a very low response rate to invitations to participate (5.47% in the full sample of ~500,000; Allen et al. 2012). We would thus expect the individuals studied here would not be fully representative of males and females from the general UK population. This was the case for education: individuals with college or university degrees were over-represented (see Method), though the male:female education ratio itself appeared representative. These selection effects may in part explain the differences between our results and those of previous studies and meta-analyses, as discussed above. Although we adjusted for the effects of age, it should also be noted—as for any study with a relatively wide age range—that there was substantial variation in the birth date of the participants, undoubtedly leading to different (unmeasured) social experiences during their development.

On the topic of age adjustment, it should also be noted that we adjusted for linear effects of age, whereas some variables may have nonlinear trends (although, given a preliminary analysis as described in the Supplemental Materials, we would not expect this to affect the sex differences in these variables to a substantial extent). We should also note that, as described in the Supplementary Materials, there was a significant, yet small, age difference between the sexes (men were older by 1.27 years on average). A final issue of representativeness concerns clinical outcomes. Although we noted above that there is much interest in sex-differential patterns of psychiatric disorder diagnoses, the unrepresentativeness of UK Biobank extends to generally low rates of such disorders in general in the sample. For this reason, we did not attempt to link the MRI sex differences observed here to clinical diagnoses, though studies of normal-range variation in traits linked to psychiatric disease (such as neuroticism, a known risk factor for Major Depressive Disorder; Kotov et al. 2010), may produce more fruitful results.

Caution should be taken in interpreting the results of the analyses involving the cognitive tests (the mediation analyses in addition to the correlations). Whereas previous, representative studies (e.g., Johnson et al. 2008) have found no mean difference, but a variance difference, in general cognitive test performance, the tests examined here showed mean differences but no strong variance differences. This may be due to problems of sample representativeness (Dykiert et al. 2009), or due to the tests tapping specific cognitive skills rather than general ability (Burgaleta et al. 2012). The cognitive measures were relatively psychometrically poor compared to a full IQ assessment: the verbal-numerical reasoning test had only 13 items, and the reaction time test had only 4 trials that counted towards the final score (see Lyall et al. 2016, for analyses of the reliability of these tests). Although the tests—particularly verbal-numerical reasoning—have some external validity (Hagenaars et al. 2016), the above issues mean that the cognitive analyses reported here should be considered preliminary. Fuller cognitive testing, currently underway in UK Biobank, will allow a more comprehensive exploration. Studies that use tests where males or females are known to show higher average scores (such as 3D mental rotation tests, which generally show higher scores in males; Maeda and Yoon 2013), would potentially allow for more informative results. In addition, cross-sectional mediation models of observational data, such as those used here, are inherently limited: they cannot address causal relations between variables. The models were simple, including only 3 main variables (sex, the brain measure, and cognitive ability; Fig. S11). Note also that there exists a great deal of debate over testing the quantifying the indirect effect in mediation models (e.g., Hayes and Scharkow 2013). More complex models, using longitudinal data and latent variables derived from multiple cognitive tests, should be specified in future research.

Finally, although this study used a wide variety of neuroimaging measures, it should be noted that these were but a small selection of the possible modalities that we could have investigated, and that studies should address in future. Other diffusion and NODDI measures of white matter microstructure such as radial and axial diffusivity and intracellular volume fraction (Cox et al. 2016), cortical measures such as regional gyrification (Gregory et al. 2016) and grey matter density (Ruigrok et al. 2014), and pathological brain structures such as white matter hyperintensities (Wardlaw et al. 2015) and enlarged perivascular spaces (Potter et al. 2015) may show interesting patterns of sex differences both across the population, and in how they relate to healthy behavioral variation as well as disease states.

## Conclusions

The present study is the largest single-sample study of neuroanatomical sex differences to date. We report evidence on the pattern of sex differences in brain volume, surface area, cortical thickness, white matter microstructure, and functional connectivity between adult males and females in the range between middle- and older-age. As has previously been argued (Fine 2017), providing a clear characterization of neurobiological sex differences is a step towards understanding patterns of differential prevalence in neurodevelopmental disorders such as autism spectrum disorder (Baron-Cohen et al. 2011), a variety of psychiatric conditions such as schizophrenia (Aleman et al. 2003), and neurodegenerative disorders such as Alzheimer’s Disease (Viña and Lloret 2010; Mazure and Swendsen 2016). We hope that the results provided here, given their large-scale, multimodal nature, will constitute an authoritative point of reference for future studies on a wide range of questions on brain sex differences. Insights into how and where the brain differs as a function of sex—with considerably more precision than in previous investigations—will enable more targeted examinations into potential drivers of these differences across psychiatric, psychological, and other domains. In particular, integrating macrostructural, microstructural, and functional data is an important long-term goal (Gur and Gur 2017). Data on many thousands of further MRI scans (to a maximum sample of 100,000 with MRI data) will be available from UK Biobank in the coming years, in addition to more complex cognitive testing batteries and genotypic data. Future studies will be able to explore in much greater depth the links between sex differences in the brain, their possible causes, and their potential medical and behavioral consequences.

## Supplementary Material

Supplementary Data
