# Supplementary material for: Sex Differences in the Adult Human Brain: Evidence from 5216 UK Biobank Participants
Source: Cereb Cortex. 2018 May 16;28(8):2959–75. doi: 10.1093/cercor/bhy109 (PMC6041980; doi:10.1093/cercor/bhy109)

Supplemental Materials for:

**Sex differences in the adult human brain:**

**Evidence from 5,216 UK Biobank participants**

Stuart J. Ritchie *et al.*

*Centre for Cognitive Ageing and Cognitive Epidemiology*

*The University of Edinburgh*

**Contents:**

Supplemental Materials and Methods

Supplemental References

Supplemental Tables S1-S14*

Supplemental Figures S1-S13

*Note: Tables S2, S3, S4, S9, and S14 are downloadable as Excel spreadsheets from this manuscript’s associated Open Science Framework page, at the following URL: <https://osf.io/k5jsn/>

**Supplemental Materials and Methods**

**Participants**

*Demographics and representativeness.* To ascertain sample representativeness, we compared the sexes on various indicators of socioeconomic status. There was no significant difference in the reported neighbourhood deprivation level, measured by Townsend Deprivation Index (Townsend, 1987), between males and females (*t*(5134.4) = 0.75, *p* = .45, *d* = 0.02). Females reported a significantly lower household income (linear-by-linear association test *z* = −6.24, *p* = 4.47×10^-10^). Note that these two measures were for neighbourhood and household, respectively, so contained additional noise in comparison to personal measures; no personal occupation or income measure was available in UK Biobank.

A personal measure of socioeconomic status is educational attainment. We compared the distributions of educational attainment in UK Biobank to the data from the 2011 Census for England and Wales (available at the following URL: <https://www.nomisweb.co.uk/census/2011>; England and Wales makes up the vast majority—around 89%—of the population of the United Kingdom). In the census, for those ages 50+ years, 25.5% of males and 20.5% of females reported having a ‘level 4 qualification’, the category including college/university degrees (we might expect this figure to be slightly higher were it restricted to the 44-77 age group, but that precise age subset was not available from the census data). In the subsample of UK Biobank used here, 48.0% of males and 42.2% of females reported having a college degree. Thus, the sample was not representative in terms of educational attainment: a higher proportion of individuals in general had a degree. However, it was not differentially representative by sex: there was around the same percentage-point difference between the sexes in college degree attainment.

91.2% of participants in this UK Biobank subsample reported their ethnic background as White, 3.6% as Mixed, 3.5% as Asian, 0.8% as Black, and 0.5% as Other. The corresponding percentages for the age group 45-74 in the 2011 Census of England and Wales were: 92.0% White, 0.7% Mixed, 4.4% Asian, 2.2% Black, and 0.6% Other.

*Age.* Females had a mean age of 61.12 years (SD = 7.42, range = 44.64-77.12 years), and males had a mean age of 62.39 years (SD = 7.56, range = 44.23-76.99 years). Males were statistically significantly older than females, though the mean difference (1.27 years) was small in effect size (*t*(5128.6) = −6.15, *p* = 8.11×10^-10^, *d* = −0.17).

*Height.* The females in the study had a mean height of 162.80cm (SD = 6.25), and the males had a mean height of 176.31cm (SD = 6.43). This mean difference (13.51cm) was strongly significant and very large in effect size (*t*(4920.50) = −75.35, *p* = ~0.00, *d* = −2.15).

**Statistical power**

In a two-sample *t*-test, groups of 2,400 participants each are enough to detect differences as low as *d* = 0.08 with 80% power. A sample size of 2,400 participants is also enough to detect correlations as low as *r* = .06 with 80% power.

**Cognitive tests**

*Verbal-numerical reasoning.* This test (UK Biobank data field 20016) consisted of thirteen multiple-choice items, six verbal and seven numerical. Participants responded to the items on a touch-screen computer. One of the verbal items was: “Stop means the same as: Pause/Close/Cease/Break/Rest/Do not know/Prefer not to answer”. One of the numerical items was: “If sixty is more than half of seventy-five, multiply twenty-three by three. If not subtract 15 from eighty-five. Is the answer: 68/69/70/71/72/Do not know/Prefer not to answer”. Participants had a two-minute time limit to answer the thirteen questions. The “prefer not to answer” option was considered as missing data for the purposes of the present analyses. The scores from the test formed a normal distribution.

*Reaction Time.* This test (UK Biobank data field 100032), which followed immediately after the verbal-numerical reasoning test, was modelled on the game of ‘snap’: participants responded by pressing a button on a button box as quickly as possible with their dominant hand whenever the symbols on two ‘cards’ displayed to them on the computer screen matched. The test had twelve rounds; the first four rounds were considered ‘training’ (or practice) rounds so were not included in the calculation of the final score, and four of the remaining rounds did not include matching symbols. Thus, the final score was calculated on the basis of the four rounds with matching symbols (the mean time in ms to press the button across these four trials was the score variable). We excluded the scores of 8 participants who had Reaction Times of 1100ms or longer. After this exclusion, the Reaction Times formed an approximately normal distribution. Note that, for analyses, we reflected the raw scores so that higher scores meant better performance (this meant that the two cognitive tests correlated positively with each other).

Note that the participants also completed three other cognitive tests at the same visit as the two described above. These tests (prospective memory, pairs matching, and numeric memory) either showed strong ceiling effects or had problems with their administration, and we made the decision not to analyse them in the present study.

**Statistical Analysis**

*Adjustment methods.* To control the neurostructural measures for age and ethnicity, total brain volume, and height, we computed a linear regression model (ordinary least squares) with the brain variable as the dependent variable and the controls as predictors, then saved the residuals. Note that we used total brain volume instead of intracranial volume, as is used in some previous studies (e.g. Muller et al., 2016), since the intracranial volume shows only minimal change with age (Royle et al., 2013) and thus produces an approximation of brain atrophy across the lifespan; such an analysis was not the objective of the current study. There are different methods used in the literature to correct smaller brain structures for total brain volume, with some researchers using a proportion and others, like us, using a regression-residuals method (O’Brien et al., 2011). In these data, we found that the proportion calculated from each subcortical volume was still (negatively) correlated with total brain volume, indicating that the proportion method, as expected (Giedd et al., 2012), had not removed all variance that was related to total brain volume (range *r* = −.35 to −.16). For that reason, we used the residual method as opposed to the proportion method to adjust for total brain volume.

*Collinearity.* Since sex and total brain volume, and sex and height, are strongly related, we ran a set of models including age, ethnicity, sex, and either total brain volume or height predicting each subcortical volume. The variance inflation factors (VIFs) were all well within acceptable limits (for total brain volume adjustment, VIF range = 1.10-2.93; for height adjustment, VIF range = 1.06-1.69). Thus, there was no indication of unacceptable collinearity in these models. For the analyses of age-by-sex interaction, we also used linear regression modelling, specifying main effects of age, sex, and their interaction, along with the covariate of ethnicity, in predicting each brain measure.

We considered whether there were nonlinear age effects in the sample, by checking whether a subset of variables (the overall and subcortical brain variables) could be predicted by age squared in addition to age in a series of regression models. We found that most could not; the only variables that had a significant contribution to their variance from age-squared were the left and right hippocampus, the left and right nucleus accumbens, and the right caudate nucleus. For these variables, the sex difference when controlling for age alone did not differ substantially from the sex difference when controlling for age and age squared (average difference in Cohen’s *d* across all five variables = 0.006). For this reason, we decided not to include controls for nonlinear effects of age in our analyses.

*Bayes Factor analysis.* We calculated Bayes Factors (BF_10_ values) for each comparison. These values indicate the probability of the alternative hypothesis (in this case that there is a sex difference) compared to that of the null hypothesis (in this case that there is no sex difference). For example, a BF_10_ value of 2 would indicate that the hypothesis of a sex difference is twice as likely (2/1 = 2) than the hypothesis of no sex difference. Conversely, BF_10_ values under 1 indicate a higher likelihood of the null hypothesis than the alternative hypothesis. For example, a BF_10_ value of 0.5 indicates that the null hypothesis of no sex difference is twice as likely (1/0.5 = 2) as the hypothesis that there is a difference.

*Head position.* In a supplementary analysis, we examined the degree to which head positioning in the scanner affected our results. To do this, we residualized the variables not just for age and ethnicity, but also for four head positioning variables—the lateral (X), transverse (Y), and longitudinal (Z) co-ordinates of the head in the scanner, and the scanner table position (UK Biobank data fields 25756-25759)—and ran the same sex differences analyses as shown in Table 1 in the main document. Results are shown in the rightmost part of Table S1, and show that adjusting for head positioning made only a negligible difference to our results.

*Mediation models.* To assess whether the sex difference in cognitive test score was mediated by brain structural measures, we tested whether the ‘indirect’ path in the model (the paths labelled *β*_sex-mediator_ and *β*_mediator-cog_ in Figure S11) was significant. To assess the significance of the indirect, mediating path, we used bootstrapped 95% confidence intervals with 1,000 iterations. To estimate the proportion of mediation, we used the following simple equation from Iacobucci et al. (2007):

$$\frac{(a\times b)}{\left( a\times b \right)+c}$$

(1)

Where *a* is the estimate for the relation between sex and the mediator (*β*_sex-mediator_ in Figure S11), *b* is the estimate for the relation between the mediator and cognitive ability (*β*_mediator-cog_), and and *c* is the ‘direct’ relation between sex and cognitive ability (*β*_sex-cog_). The formula thus produces an estimate of the proportion of the total variance that is hypothesized to run through the ‘indirect’ path. Proportions were converted to percentages. Mediation models were run using the *lavaan* package for R (Rosseel, 2012). Note that the models using brain macrostructure as a mediator (that is, the brain volume measures, surface area, and cortical thickness), each contained only three variables; they were thus saturated and the standard absolute fit indices for structural equation models (Root Mean Square Error of Approximation, Comparative Fit Index, etc.) could not be computed. For the models using brain microstructure (fractional anisotropy and orientation dispersion), we added some residual covariance paths to improve the model fit, as indicated by *lavaan*’s modification indices. These did not result in substantial changes to the estimated parameters in the models; the fit statistics—Root Mean Square Error of Approximation (RMSEA), Comparative Fit Index (CFI), Tucker-Lewis Index (TLI), and Standardized Root Mean Square Residual (SRMR)—are reported in the notes to Tables S10 and S11. However, measurement invariance tests (using the *measurementInvariance* function of the *semTools* package for R; semTools Contributors, 2016), assessing whether the same latent construct was being measured across the sexes (e.g. Widaman et al., 2010) indicated that strong measurement invariance could not be assumed for either variable. That is, models with the factor loadings and intercepts set to equality across the sexes—strong invariance models—fit significantly more poorly, by the *χ*^2^ test, than configural invariance models where these parameters were freely-estimated (for general fractional anisotropy, comparing configural and strong invariance models, Δ*χ*^2^(11) = 766.46, *p* < .001; for general orientation dispersion: Δ*χ*^2^(11) = 481.11, *p* < .001). Thus, the results for the two latent brain microstructural mediators need to be assessed with caution.

Finally, we ran multiple-mediator models assessing which brain subregions, parcellated in terms of the Desikan-Killiany atlas, were most important in mediating the sex-cognitive relation. We did so for volume, surface area, and cortical thickness as mediators, and for verbal-numerical reasoning and reaction time as outcome measures. Note that, since so many brain regions were included, there was a substantial degree of missingness, meaning that the complete-cases sample (a requirement for the LASSO model described below) was very small. We used Multiple Imputation by Chained Equations (MICE, using the *mice* package for R; van Buuren & Groothuis-Oudshoorn, 2011) to impute the missing datapoints. Further, since there were many, strongly-correlated variables across the multiple brain regions, we used two techniques to reduce the numbers of variables included in the regression model. First, we averaged each measure across the brain hemispheres. Second, we used a least absolute shrinkage and selection operator (LASSO) regression model (Tibshirani, 1996) in the *glmnet* package for R (Friedman et al., 2010) to produce a sparser selection of the brain variables that were most relevant for explaining variation in the cognitive test score in one randomly-selected half of the sample. After 10-fold cross-validation, we used the shrinkage parameter that minimised the mean-` error (λ_min_). Cross-validation and coefficient shrinkage plots for each model are provided in Figure S12. We used each of the selected variables as mediators in a model on the second randomly-selected half of the sample (that is, the variables were selected and tested in different datasets). We allowed for covariances between the mediators, and used the same methods described above to estimate the percentage of the association between sex and the cognitive test score that was mediated by the volume, surface area, or thickness of each brain region. These percentages are provided in Table S12 and illustrated in Figure 4.

**Supplemental References**

Friedman J, Hastie T, Tibshirani R. 2010. Regularization paths for generalized linear models via coordinate descent. J Stat Softw. 39:1-13.

Giedd JN, Raznahan A, Mills KL, Lenroot RK. 2012. Magnetic resonance imaging of male/female differences in human adolescent brain anatomy. Biol Sex Differ. 3:19.

Iacobucci, D., Saldanha, N., & Deng, X. A meditation on mediation: Evidence that structural equations models perform better than regressions. J. Consum. Psychol. 17, 139-153 (2007).

Muller, M. Sigurdsson S, Kjartansson O, Gunnarsdottir I, Thorsdottir I, Harris TB, van Buchem M, Gudnason V, Launer LJ, Age, Gene/Environment Susceptibility-Reykjavik Study Investigators. 2016. Late-life brain volume: a life-course approach. The AGES-Reykjavik study. Neurobiol Aging. 41:86-92.

O'Brien LM, Ziegler DA, Deutsch CK, Frazier JA, Herbert MR, Locascio JJ. 2011. Statistical adjustments for brain size in volumetric neuroimaging studies: some practical implications in methods. Psychiatr Res Neuroimaging. 193:113-122.

Rosseel Y. 2012. lavaan: An R Package for Structural Equation Modeling. J Stat Soft. 48:1-36.

Royle NA, Booth T, Hernández MC, Penke L, Murray C, Gow AJ, Maniega SM, Starr J, Bastin ME, Deary IJ, Wardlaw JM. 2013. Estimated maximal and current brain volume predict cognitive ability in old age. Neurobiol Aging. 34:2726-2733.

semTools Contributors. 2016. semTools: Useful tools for structural equation modelling. R package v.0.4-14. URL: <https://CRAN.R-project.org/package=semTools>.

Tibshirani R. 1996. Regression shrinkage and selection via the lasso. J Royal Stat Soc B. 58:267-288.

Townsend P. 1987. Deprivation. J Soc Policy 16:125-146.

van Buuren S, Groothuis-Oudshoorn K. 2011. mice: Multivariate Imputation by Chained Equations in R. J Stat Soft. 45:1-67.

Widaman KF, Ferrer E, Conger RD. 2010. Factorial invariance within longitudinal structural equation models: Measuring the same construct across time. Child Dev Perspect. 4:10-18.

**Supplemental Tables**

*Table S1.* Mean and variance differences in subcortical volumes after adjustment for total brain volume and height (all variables also adjusted for age and ethnicity).

| Brain volume | Adjusting for total brain volume | | | | | | Adjusting for height | | | | | | Adjusting for head position | | | | | |
| --- | --- | --- | --- | --- | --- | --- | --- | --- | --- | --- | --- | --- | --- | --- | --- | --- | --- | --- |
|  | Mean difference test | | | | Variance Ratio test | | Mean difference test | | | | Variance Ratio test | | Mean difference test | | | | Variance Ratio test | |
|  | *t* | *p*_adj_ | *d* | BF_10_ | VR | *p*_adj_ | *t* | *p*_adj_ | *d* | BF_10_ | VR | *p*_adj_ | *t* | *p*_adj_ | *d* | BF_10_ | VR | *p*_adj_ |
| Total brain | - | - | - | - | - | - | −14.40 | 4.76×10^-46^ | −0.42 | 9.66×10^42^ | 0.84 | 1.87×10^-05^ | −44.80 | ~0.00 | −1.29 | 1.87×10^368^ | 0.82 | 9.72×10^-06^ |
| Grey matter | - | - | - | - | - | - | −10.50 | 1.72×10^-25^ | −0.31 | 2.41×10^22^ | 0.83 | 4.33×10^-06^ | −37.01 | 8.75×10^-263^ | −1.07 | 4.56×10^263^ | 0.83 | 2.85×10^-05^ |
| White matter | - | - | - | - | - | - | −16.00 | 4.26×10^-56^ | −0.47 | 1.35×10^53^ | 0.83 | 8.66×10^-06^ | −46.43 | ~0.00 | −1.34 | 1.94×10^391^ | 0.82 | 1.42×10^-05^ |
| Left hippocampus | −0.77 | 1.00 | −0.02 | 0.04 | 0.86 | 4.30×10^-04^ | −5.77 | 2.48×10^-08^ | −0.17 | 5.93×10^5^ | 0.87 | .001 | −17.79 | 6.46×10^-68^ | −0.51 | 4.03×10^65^ | 0.86 | 3.57×10^-04^ |
| Right hippocampus | −0.42 | 1.00 | −0.01 | 0.03 | 0.74 | 5.41×10^-13^ | −5.97 | 1.05×10^-08^ | −0.18 | 2.13×10^6^ | 0.78 | 1.38×10^-08^ | −17.02 | 1.70×10^-62^ | −0.50 | 3.67×10^60^ | 0.77 | 1.83×10^-09^ |
| Left accumbens | 2.58 | .07 | 0.08 | 0.93 | 0.82 | 1.28×10^-05^ | −2.18 | .06 | −0.06 | 0.36 | 0.81 | 4.16×10^-06^ | −13.29 | 2.60×10^-39^ | −0.39 | 4.47×10^36^ | 0.81 | 1.21×10^-06^ |
| Right accumbens | 3.52 | .003 | 0.10 | 16.64 | 0.83 | 1.96×10^-05^ | −1.70 | .09 | −0.05 | 0.14 | 0.83 | 4.77×10^-05^ | −10.81 | 6.34×10^-27^ | −0.32 | 6.36×10^23^ | 0.83 | 3.13×10^-05^ |
| Left amygdala | −5.98 | 2.59×10^-08^ | −0.18 | 2.38×10^6^ | 0.73 | 1.12×10^-13^ | −6.92 | 3.12×10^-11^ | −0.21 | 1.06×10^9^ | 0.74 | 5.81×10^-12^ | −18.71 | 1.27×10^-74^ | −0.55 | 1.50×10^73^ | 0.74 | 4.87×10^-13^ |
| Right amygdala | −6.05 | 1.89×10^-08^ | −0.18 | 3.31×10^6^ | 0.79 | 2.94×10^-08^ | −6.20 | 3.00×10^-09^ | −0.18 | 8.91×10^6^ | 0.78 | 2.84×10^-08^ | −16.07 | 4.13×10^-56^ | −0.47 | 7.12×10^53^ | 0.78 | 2.25×10^-08^ |
| Left caudate | 0.33 | 1.00 | −0.01 | 0.03 | 0.86 | 3.67×10^-04^ | −6.79 | 8.93×10^-11^ | −0.20 | 3.42×10^8^ | 0.85 | 8.16×10^-04^ | −23.00 | 3.04×10^-110^ | −0.66 | 2.70×10^108^ | 0.85 | 3.17×10^-04^ |
| Right caudate | 0.05 | 1.00 | 0.001 | 0.03 | 0.83 | 2.04×10^-05^ | −6.46 | 6.96×10^-10^ | −0.19 | 3.98×10^6^ | 0.84 | 2.24×10^-04^ | −22.67 | 2.38×10^-107^ | −0.65 | 4.08×10^105^ | 0.84 | 4.91×10^-05^ |
| Left pallidum | −5.51 | 3.69×10^-07^ | −0.16 | 1.26×10^5^ | 0.92 | .04 | −8.13 | 3.76×10^-15^ | −0.24 | 6.50×10^12^ | 0.91 | .03 | −24.64 | 1.98×10^-125^ | −0.71 | 3.45×10^123^ | 0.89 | 2.66×10^-03^ |
| Right pallidum | −0.12 | 2.82×10^-04^ | −0.12 | 196.54 | 0.87 | 8.09×10^-04^ | −8.20 | 2.44×10^-15^ | −0.24 | 1.30×10^13^ | 0.86 | 6.12×10^-04^ | −25.18 | 1.79×10^-130^ | −0.73 | 9.20×10^128^ | 0.85 | 3.17×10^-04^ |
| Left putamen | −7.63 | 3.58×10^-13^ | −0.22 | 1.56×10^11^ | 0.83 | 2.80×10^-05^ | −10.23 | 2.88×10^-23^ | −0.30 | 1.74×10^21^ | 0.82 | 9.50×10^-06^ | −31.38 | 1.26×10^-263^ | −0.91 | 2.06×10^195^ | 0.84 | 5.91×10^-05^ |
| Right putamen | −8.63 | 1.16×10^-16^ | −0.25 | 5.28×10^14^ | 0.81 | 6.04×10^-07^ | −11.79 | 1.63×10^-30^ | −0.35 | 3.95×10^28^ | 0.81 | 1.76×10^-06^ | −33.64 | 9.63×10^-222^ | −0.97 | 9.77×10^221^ | 0.83 | 2.08×10^-05^ |
| Left thalamus | 0.28 | .78 | 0.01 | 0.03 | 0.75 | 9.65×10^-12^ | −8.71 | 3.80×10^-17^ | −0.26 | 9.58×10^14^ | 0.85 | 3.21×10^-04^ | −31.76 | 7.65×10^-200^ | −0.92 | 7.06×10^199^ | 0.82 | 1.49×10^-05^ |
| Right thalamus | −0.56 | .58 | −0.02 | 0.04 | 0.77 | 3.22×10^-10^ | −9.32 | 1.83×10^-19^ | −0.27 | 2.10×10^17^ | 0.85 | 4.48×10^-04^ | −33.73 | 7.34×10^-223^ | −0.97 | 9.42×10^222^ | 0.83 | 4.91×10^-05^ |

*Note:* Negative *t*/*d*-values indicate higher male mean. VR = Variance ratio (values < 1 indicate greater male variance). *p*_adj_-values adjusted for False Discovery Rate. BF_10_ = Bayes Factor indicating the probability of the hypothesis (that there is a sex difference) compared to the null hypothesis (that there is no sex difference). Tests were not performed adjusting total, grey, and white matter volumes for total brain volume as these variables were highly collinear (or identical) to the variable being adjusted for.

*Table S2.* Full results of sex differences in volume, surface area, and cortical thickness (not controlled for total brain volume, but controlled for age and ethnicity) across the regions of the Desikan-Killiany neuroanatomical atlas.

See Excel spreadsheet available at the Open Science Framework. Direct link: <https://osf.io/w3u8e/>

*Table S3.* Full results for sex differences in volume, surface area, and cortical thickness (adjusted – controlled for total brain volume, in addition to age and ethnicity) across the regions of the Desikan-Killiany neuroanatomical atlas.

See Excel spreadsheet available at the Open Science Framework. Direct link: <https://osf.io/4gwdv/>

*Table S4.* Full results for sex differences in volume, surface area, and cortical thickness (adjusted – controlled for height, in addition to age and ethnicity) across the regions of the Desikan-Killiany neuroanatomical atlas.

See Excel spreadsheet available at the Open Science Framework. Direct link: <https://osf.io/798bn/>

*Table S5*. Descriptive statistics for sex differences (in mean and in variance) in fractional anisotropy (FA) in 22 white matter tracts.

| Tract | Female  (*n* = 2,750) | Male  (*n* = 2,466) | Mean difference test | | | | Variance Ratio test | | Mean difference test (TBV adjusted) | | | | Variance Ratio test (TBV adjusted) | |
| --- | --- | --- | --- | --- | --- | --- | --- | --- | --- | --- | --- | --- | --- | --- |
|  | M FA (SD) | M FA (SD) | *t* | *p*_adj_ | *d* | BF_10_ | VR | *p*_adj_ | *t* | *p*_adj_ | *d* | BF_10_ | VR | *p*_adj_ |
| Left Arcuate | .423 (.021) | .426 (.023) | −5.48 | 4.91×10^-07^ | −0.17 | 1.19×10^05^ | 0.87 | .14 | −0.99 | 1.00 | −0.03 | 0.06 | 0.87 | .02 |
| Right Arcuate | .410 (.020) | .420 (.021) | −16.56 | 2.27×10^-58^ | −0.51 | 1.83×10^56^ | 0.95 | 1.00 | −8.46 | 8.38×10^-16^ | −0.26 | 8.62×10^13^ | 0.95 | 1.00 |
| Left ATR | .400 (.018) | .403 (.018) | −6.22 | 6.54×10^-09^ | −0.19 | 8.13×10^06^ | 0.93 | .95 | −0.53 | 1.00 | −0.02 | 0.04 | 0.92 | .58 |
| Right ATR | .392 (.017) | .396 (.018) | −8.55 | 2.48×10^-16^ | −0.27 | 2.14×10^14^ | 0.91 | .39 | −0.92 | 1.00 | −0.03 | 0.05 | 0.90 | .20 |
| Left Cingulum | .533 (.034) | .544 (.033) | −11.83 | 1.42×10^-30^ | −0.36 | 2.12×10^28^ | 1.02 | 1.00 | −3.40 | 9.65×10^-03^ | −0.10 | 10.75 | 1.00 | 1.00 |
| Right Cingulum | .495 (.032) | .503 (.033) | −8.81 | 2.95×10^-17^ | −0.27 | 1.75×10^15^ | 0.94 | .95 | −3.05 | .03 | −0.09 | 3.64 | 0.93 | .83 |
| Left CST | .544 (.022) | .552 (.021) | −12.64 | 1.18×10^-34^ | −0.39 | 1.95×10^32^ | 1.09 | .48 | −4.85 | 2.47×10^-05^ | −0.15 | 3.85×10^03^ | 1.07 | .82 |
| Right CST | .536 (.023) | .547 (.021) | −17.53 | 4.10×10^-65^ | −0.54 | 1.39×10^62^ | 1.17 | 5.33×10^-03^ | −7.27 | 8.93×10^-12^ | −0.22 | 6.42×10^09^ | 1.15 | .02 |
| Forceps Major | .583 (.026) | .584 (.028) | −1.20 | .93 | −0.04 | 0.07 | 0.85 | 5.33×10^-03^ | 1.02 | 1.00 | 0.03 | 0.06 | 0.85 | 4.48×10^-03^ |
| Forceps Minor | .466 (.020) | .468 (.021) | −4.89 | 1.06×10^-05^ | −0.15 | 5.60×10^03^ | 0.85 | 3.47×10^-03^ | −0.12 | 1.00 | −0.003 | 0.03 | 0.86 | 5.14×10^-03^ |
| Left IFOF | .478 (.021) | .477 (.022) | 1.35 | .88 | 0.04 | 0.09 | 0.83 | 5.89×10^-04^ | 3.84 | 2.04×10^-03^ | 0.12 | 5.67 | 0.83 | 4.86×10^-04^ |
| Right IFOF | .466 (.020) | .466 (.021) | −1.15 | .93 | −0.04 | 0.07 | 0.86 | 5.42×10^-03^ | 1.65 | 1.00 | 0.05 | 0.14 | 0.85 | 3.46×10^-03^ |
| Left ILF | .463 (.019) | .461 (.020) | 3.26 | 8.84×10^-03^ | 0.10 | 7.24 | 0.92 | .49 | 4.52 | 1.07×10^-04^ | 0.14 | 968.16 | 0.89 | .07 |
| Right ILF | .453 (.018) | .452 (.019) | −1.09 | .93 | −0.03 | 0.06 | 0.88 | .04 | 1.66 | 1.00 | 0.05 | 0.14 | 0.85 | 3.35×10^-03^ |
| Left PTR | .460 (.019) | .458 (.021) | 3.12 | .01 | 0.10 | 4.61 | 0.82 | 7.15×10^-05^ | 3.80 | 2.20×10^-03^ | 0.12 | 50.08 | 0.82 | 7.40×10^-05^ |
| Right PTR | .454 (.019) | .455 (.021) | −2.82 | .03 | −0.09 | 1.89 | 0.86 | .01 | −0.15 | 1.00 | −0.005 | 0.03 | 0.86 | 7.31×10^-03^ |
| Left SLF | .443 (.021) | .442 (.021) | 0.12 | .93 | 0.004 | 0.03 | 0.94 | .95 | 2.84 | .05 | 0.09 | 1.94 | 0.94 | .83 |
| Right SLF | .425 (.020) | .427 (.020) | −4.36 | 1.17×10^-04^ | −0.14 | 459.92 | 0.96 | 1.00 | 1.46 | 1.00 | 0.05 | 0.10 | 0.96 | 1.00 |
| Left STR | .420 (.017) | .427 (.018) | −12.27 | 9.55×10^-33^ | −0.38 | 7.13×10^30^ | 0.87 | .02 | −4.70 | 4.80×10^-05^ | −0.15 | 2.27×10^03^ | 0.86 | 7.31×10^-03^ |
| Right STR | .419 (.018) | .426 (.019) | −12.08 | 8.74×10^-32^ | −0.38 | 6.58×10^29^ | 0.89 | .11 | −5.05 | 9.07×10^-06^ | −0.16 | 1.24×10^04^ | 0.88 | .03 |
| Left Uncinate | .390 (.024) | .395 (.024) | −8.41 | 7.63×10^-16^ | −0.26 | 5.44×10^13^ | 0.99 | 1.00 | −0.75 | 1.00 | −0.02 | 0.05 | 0.99 | 1.00 |
| Right Uncinate | .390 (.020) | .394 (.020) | −8.27 | 2.32×10^-15^ | −0.26 | 1.77×10^13^ | 0.97 | 1.00 | 0.41 | 1.00 | 0.01 | 0.04 | 0.96 | 1.00 |

*Note:* Arcuate = Arcuate Fasciculus; ATR = Anterior Thalamic Radiation; Cingulum = Cingulum Bundle; CST = Cortico-spinal Tract; IFOF = Inferior Fronto-Occipital Fasciculus; ILF = Inferior Longitudinal Fasciculus; PTR = Posterior Thalamic Radiation; SLF = Superior Longitudinal Fasciculus; STR = Superior Thalamic Radiation; Uncinate = Uncinate Fasciculus. BF_10_ = Bayes Factor indicating the probability of the alternative hypothesis (that there is a sex difference) compared to the null hypothesis (that there is no sex difference).

*Table S6.* Descriptive statistics for sex differences (in mean and in variance) in orientation dispersion (OD) in 22 white matter tracts.

| Tract | Female  (*n* = 2,750) | Male  (*n* = 2,466) | Mean difference test | | | | Variance Ratio test | | Mean difference test (TBV adjusted) | | | | Variance Ratio test (TBV adjusted) | |
| --- | --- | --- | --- | --- | --- | --- | --- | --- | --- | --- | --- | --- | --- | --- |
|  | M OD (SD) | M OD (SD) | *t* | *p*_adj_ | *d* | BF_10_ | VR | *p*_adj_ | *t* | *p*_adj_ | *d* | BF_10_ | VR | *p*_adj_ |
| Left AR | .244 (.016) | .241 (.017) | 5.60 | 6.81×10^-08^ | 0.17 | 2.13×10^5^ | 0.94 | 1.00 | 5.89 | 1.62×10^-08^ | 0.18 | 1.13×10^06^ | 0.95 | 1.00 |
| Right AR | .254 (.015) | .250 (.015) | 8.27 | 1.94×10^-15^ | 0.25 | 1.60×10^13^ | 1.02 | 1.00 | 8.93 | 7.80×10^-18^ | 0.28 | 4.10×10^15^ | 1.01 | 1.00 |
| Left ATR | .239 (.011) | .237 (.011) | 6.94 | 2.73×10^-11^ | 0.21 | 7.47×10^8^ | 1.04 | 1.00 | 7.69 | 1.32×10^-13^ | 0.24 | 1.62×10^11^ | 1.03 | 1.00 |
| Right ATR | .245 (.012) | .241 (.012) | 10.97 | 1.93×10^-26^ | 0.34 | 1.32×10^24^ | 1.08 | 1.00 | 11.89 | 6.68×10^-31^ | 0.37 | 3.57×10^28^ | 1.06 | 1.00 |
| Left Cingulum | .132 (.018) | .129 (.017) | 7.34 | 1.76×10^-12^ | 0.23 | 1.10×10^10^ | 1.13 | .10 | 6.79 | 6.59×10^-11^ | 0.21 | 2.41×10^08^ | 1.11 | .24 |
| Right Cingulum | .146 (.020) | .144 (.020) | 2.74 | 6.24×10^-03^ | 0.08 | 1.45 | 0.96 | 1.00 | 2.41 | 1.59×10^-02^ | 0.07 | 0.63 | 0.95 | 1.00 |
| Left CST | .160 (.014) | .153 (.013) | 16.62 | 8.02×10^-59^ | 0.51 | 1.91×10^56^ | 1.05 | 1.00 | 16.89 | 1.08×10^-60^ | 0.52 | 1.57×10^58^ | 1.04 | 1.00 |
| Right CST | .161 (.013) | .153 (.013) | 18.70 | 1.08×10^-73^ | 0.57 | 7.14×10^70^ | 1.11 | .33 | 19.11 | 8.28×10^-77^ | 0.59 | 1.12×10^74^ | 1.08 | 1.00 |
| Forceps Major | .129 (.011) | .125 (.010) | 13.12 | 2.44×10^-37^ | 0.40 | 9.33×10^34^ | 1.05 | 1.00 | 13.46 | 3.16×10^-39^ | 0.41 | 6.71×10^36^ | 1.06 | 1.00 |
| Forceps Minor | .198 (.010) | .195 (.010) | 8.23 | 2.38×10^-15^ | 0.26 | 1.40×10^13^ | 0.95 | 1.00 | 8.08 | 6.73×10^-15^ | 0.25 | 4.06×10^12^ | 0.95 | 1.00 |
| Left IFOF | .166 (.010) | .165 (.010) | 3.53 | 8.34×10^-04^ | 0.11 | 17.78 | 0.91 | .61 | 3.73 | 3.94×10^-04^ | 0.12 | 35.90 | 0.92 | .70 |
| Right IFOF | .171 (.010) | .169 (.010) | 7.79 | 6.47×10^-14^ | 0.24 | 3.70×10^11^ | 1.03 | 1.00 | 8.23 | 2.16×10^-15^ | 0.25 | 1.17×10^13^ | 1.03 | 1.00 |
| Left ILF | .183 (.008) | .181 (.008) | 5.81 | 2.76×10^-08^ | 0.18 | 7.20×10^5^ | 0.89 | .14 | 5.75 | 2.83×10^-08^ | 0.18 | 5.31×10^05^ | 0.89 | .15 |
| Right ILF | .187 (.008) | .184 (.008) | 10.83 | 8.72×10^-26^ | 0.33 | 3.16×10^23^ | 1.04 | 1.00 | 10.83 | 8.87×10^-26^ | 0.33 | 3.09×10^23^ | 1.05 | 1.00 |
| Left PTR | .184 (.009) | .181 (.009) | 10.30 | 2.03×10^-23^ | 0.32 | 1.76×10^21^ | 0.97 | 1.00 | 10.67 | 4.49×10^-25^ | 0.33 | 7.61×10^22^ | 0.97 | 1.00 |
| Right PTR | .187 (.010) | .183 (.010) | 12.72 | 3.63×10^-35^ | 0.39 | 6.43×10^32^ | 1.05 | 1.00 | 13.35 | 1.33×10^-38^ | 0.41 | 1.55×10^36^ | 1.06 | 1.00 |
| Left SLF | .225 (.012) | .222 (.012) | 6.68 | 1.36×10^-10^ | 0.21 | 1.37×10^8^ | 1.01 | 1.00 | 6.90 | 3.65×10^-11^ | 0.21 | 5.89×10^08^ | 1.01 | 1.00 |
| Right SLF | .234 (.012) | .231 (.012) | 8.16 | 4.09×10^-15^ | 0.25 | 5.82×10^12^ | 1.07 | 1.00 | 8.48 | 3.03×10^-16^ | 0.26 | 8.30×10^13^ | 1.06 | 1.00 |
| Left STR | .252 (.014) | .244 (.016) | 18.16 | 1.41×10^-69^ | 0.57 | 1.39×10^68^ | 0.82 | 1.06×10^-04^ | 18.93 | 2.84×10^-75^ | 0.60 | 1.38×10^74^ | 0.78 | 3.71×10^-07^ |
| Right STR | .251 (.014) | .243 (.016) | 17.41 | 3.20×10^-64^ | 0.55 | 6.98×10^62^ | 0.80 | 3.32×10^-06^ | 18.20 | 7.40×10^-70^ | 0.58 | 6.43×10^68^ | 0.75 | 1.90×10^-09^ |
| Left Uncinate | .236 (.016) | .231 (.015) | 9.18 | 8.82×10^-19^ | 0.28 | 2.97×10^16^ | 1.11 | .27 | 9.04 | 3.12×10^-18^ | 0.28 | 8.52×10^15^ | 1.11 | .28 |
| Right Uncinate | .230 (.015) | .226 (.014) | 8.83 | 1.84×10^-17^ | 0.27 | 1.49×10^15^ | 1.08 | 1.00 | 8.84 | 1.50×10^-17^ | 0.27 | 1.68×10^15^ | 1.08 | 1.00 |

*Note:* AR = Acoustic Radiation; ATR = Anterior Thalamic Radiation; Cingulum = Cingulum Bundle; CST = Cortico-spinal Tract; IFOF = Inferior Fronto-Occipital Fasciculus; ILF = Inferior Longitudinal Fasciculus; PTR = Posterior Thalamic Radiation; SLF = Superior Longitudinal Fasciculus; STR = Superior Thalamic Radiation; Uncinate = Uncinate Fasciculus.

*Table S7.* Correlation matrix for overall brain variables and cognitive variables in the full sample (matrices for random sample halves shown in Table S8).

| Variable | 1. | 2. | 3. | 4. | 5. | 6. | 7. | 8. | 9. |
| --- | --- | --- | --- | --- | --- | --- | --- | --- | --- |
| 1. Total brain volume | - |  |  |  |  |  |  |  |  |
| 2. Grey matter volume | .941 | - |  |  |  |  |  |  |  |
| 3. White matter volume | .958 | .805 | - |  |  |  |  |  |  |
| 4. Total surface area | .910 | .856 | .874 | - |  |  |  |  |  |
| 5. Mean cortical thickness | −.016^†^ | .168 | −.174 | −.228 | - |  |  |  |  |
| 6. General fractional anisotropy | .180 | .204 | .141 | .120 | .172 | - |  |  |  |
| 7. General orientation dispersion | −.435 | −.425 | −.401 | −.420 | −.065 | −.216 | - |  |  |
| 8. Verbal-numerical reasoning | .177 | .175 | .159 | .166 | .033^†^ | .045 | −.077 | - |  |
| 9. Reaction time | .099 | .074 | .107 | .081 | .001^†^ | .025^†^ | −.012^†^ | .122 | - |
| 10. Sex (female=0, male=1) | −.571 | −.484 | −.591 | −.566 | .150 | −.114 | .286 | −.089 | −.108 |

Note: ^†^ = not statistically significant (*p* > .05). All other correlations significant at *p* < .05.

*Table S8.* Correlation matrix for overall brain variables and cognitive variables in the two randomly selected halves of the sample (below diagonal: first half; above diagonal: second half).

| Variable | 1. | 2. | 3. | 4. | 5. | 6. | 7. | 8. | 9. | 10. |
| --- | --- | --- | --- | --- | --- | --- | --- | --- | --- | --- |
| 1. Total brain volume | - | .943 | .960 | .913 | −.004^†^ | .184 | −.425 | .191 | .098 | −.565 |
| 2. Grey matter volume | .940 | - | .812 | .857 | .183 | .257 | −.416 | .188 | .073 | −.470 |
| 3. White matter volume | .956 | .798 | - | .881 | −.163 | .207 | −.394 | .171 | .106 | −.589 |
| 4. Total surface area | .907 | .856 | .865 | - | −.225 | .202 | −.422 | .156 | .072 | −.556 |
| 5. Mean cortical thickness | −.029^†^ | .151 | −.186 | −.232 | - | .095 | −.105 | .024^†^ | −.003^†^ | .152 |
| 6. General fractional anisotropy | .176 | .201 | .136 | .139 | .089 | - | −.203 | .039^†^ | .023^†^ | −.113 |
| 7. General orientation dispersion | −.446 | −.434 | −.409 | −.417 | −.022^†^ | −.229 | - | −.076 | −.013^†^ | .287 |
| 8. Verbal-numerical reasoning | .162 | .162 | .146 | .176 | .042^†^ | .051^†^ | −.078 | - | .109 | −.095 |
| 9. Reaction time | .099 | .074 | .108 | .090 | .004^†^ | .028^†^ | −.014^†^ | .135 | - | −.106 |
| 10. Sex (female=0, male=1) | −.576 | −.492 | −.593 | −.577 | .149 | −.116 | .285 | −.084 | −.110 | - |

Note: ^†^ = not statistically significant (*p* > .05). All other correlations significant at *p* ≤ .001.

*Table S9.* Correlations between verbal-numerical reasoning scores, reaction times, and each brain subregion from the Desikan-Killiany Atlas for each sex, and *z*-tests of the difference between correlations across the sexes.

See Excel spreadsheet available at the Open Science Framework. Direct link: <https://osf.io/fbj37/>

*Table S10*. Tests of the hypothesized mediation of the association between sex and verbal-numerical reasoning score by individual brain measures for the two randomly-selected halves of the sample (*β* values refer to those in the path diagram shown in Figure S11).

| Sample | Mediator | *β*_sex-mediator_ (SE) | *β*_mediator-_*_VNR_* (SE) | *β_sex-VNR_* (SE) | *β*_mediation_ [95% CI] | Mediation% |
| --- | --- | --- | --- | --- | --- | --- |
| First random half (1,374 females; 1,238 males) | Total brain volume | −.578 (.013)^***^ | .174 (.025)^***^ | .021 (.025) | .113  [.082, .143] | 91.0% |
|  | Grey matter volume | −.497 (.015)^***^ | .163 (.023)^***^ | .002 (.024) | .081  [.057, .104] | 97.1% |
|  | White matter volume | −.593 (.012)^***^ | .152 (.025)^***^ | .010 (.026) | .090  [.060, .118] | 89.9% |
|  | Total surface area | −.577 (.015)^***^ | .181 (.029)^***^ | .009 (.029) | .114  [.073, .138] | 92.0% |
|  | Mean cortical thickness | .139 (.023)^***^ | .057 (.024)^*^ | −.103 (.024)^***^ | .008  [.001, .016] | 7.1% |
|  | General fractional anisotropy | −.115 (.023)^***^ | .040 (.023) | −.095 (.022)^***^ | .005  [.003, .011] | 4.7% |
|  | General orientation dispersion | .275 (.023)^***^ | −.055 (.026)^*^ | −.084 (.023) | .015  [.002, .030] | 15.3% |
| Second random half (1,376 females; 1,228 males) | Total brain volume | −.578 (.013)^***^ | .174 (.025)^***^ | .021 (.025) | .101  [.071, .130] | 82.4% |
|  | Grey matter volume | −.484 (.015)^***^ | .182 (.023)^***^ | −.012 (.023) | .088  [.064, .110] | 87.9% |
|  | White matter volume | −.595 (.012)^***^ | .172 (.025)^***^ | .002 (.025) | .102  [.070, .133] | 98.9% |
|  | Total surface area | −.559 (.015)^***^ | .162 (.028)^***^ | .010 (.028) | .090  [.058, .122] | 89.8% |
|  | Mean cortical thickness | .138 (.023)^***^ | .036 (.024) | −.085 (.024)^***^ | .005  [.0004, .012] | 5.4% |
|  | General fractional anisotropy | −.109 (.022)^**^ | .029 (.023) | −.090 (.022)^***^ | .003 [.0001, .009] | 3.4% |
|  | General orientation dispersion | .292 (.022)^***^ | −.053 (.025)^*^ | −.078 (.023)^**^ | .016 [.002, .031] | 16.6% |

*Note:* All *β*s standardized. *β*_mediation_, the ‘indirect path’ from Sex to VNR via the mediator, is with bootstrapped 95% confidence interval (1,000 iterations). VNR = Verbal-numerical reasoning test score. **p* < .05; ***p* < .01; ****p* < .001. *β_sex-VNR_* path before inclusion of the mediator for first random half: *β* = .097, SE = .020, *p* < .001; for second random half: *β* = .081, SE = .020, *p* < .001. Model fit statistics for general fractional anisotropy in first random half: RMSEA = .086, CFI = .944, TLI = .922, SRMR = .047; second random half: RMSEA = .083, CFI = .949, TLI = .930, SRMR = .044. For comparative fit index in first random half: RMSEA = .082, CFI = .899, TLI = .856, SRMR = .059; second random half: RMSEA = .085, CFI = .892, TLI = .846, SRMR = .058.

*Table S11*. Tests of the hypothesized mediation of the association between sex and reaction time by individual brain measures for the two randomly-selected halves of the sample (*β* values refer to those in the path diagram shown in Figure S11).

| Sample | Mediator | *β*_sex-mediator_ (SE) | *β*_mediator-_*_RT_* (SE) | *β_sex-RT_* (SE) | *β*_mediation_ [95% CI] | Mediation% |
| --- | --- | --- | --- | --- | --- | --- |
| First random half (1,374 females; 1,238 males) | Total brain volume | −.581 (.012)^***^ | .055 (.025)^*^ | −.076 (.025)^**^ | .032  [.005, .064] | 29.7% |
|  | Grey matter volume | −.496 (.014)^***^ | .027 (.023) | −.093 (.023)^***^ | .014  [.0001, .035] | 12.7% |
|  | White matter volume | −.597 (.012)^***^ | .069 (.025)^*^ | −.066 (.025)^**^ | .041  [.011, .073] | 38.3% |
|  | Total surface area | −.580 (.014)^***^ | .035 (.029) | −.096 (.029)^**^ | .020  [.001, .055] | 17.3% |
|  | Mean cortical thickness | .149 (.023)^***^ | .022 (.024) | −.119 (.024)^***^ | .003  [.0001, .01] | 2.7% |
|  | General fractional anisotropy | −.116 (.022)^**^ | .018 (.023) | −.092 (.022)^***^ | .002 [9.00×10^-05^, .008] | 2.2% |
|  | General orientation dispersion | .282 (.022)^***^ | .013 (.025) | −.098 (.023)^***^ | .004 [.0002, .018] | 3.5% |
| Second random half (1,376 females; 1,228 males) | Total brain volume | −.574 (.012)^***^ | .053 (.025)^*^ | −.080 (.025)^**^ | .030  [.004, .058] | 27.3% |
|  | Grey matter volume | −.484 (.015)^***^ | .028 (.023) | −.095 (.023)^***^ | .013  [.001, .035] | 12.3% |
|  | White matter volume | −.598 (.012)^***^ | .063 (.025)^*^ | −.073 (.025)^**^ | .037  [.008, .068] | 33.8% |
|  | Total surface area | −.562 (.015)^***^ | .026 (.028) | −.082 (.028)^**^ | .015  [.001, .046] | 15.2% |
|  | Mean cortical thickness | .142 (.023)^**^ | .011 (.024) | −.098 (.023)^***^ | .002  [.0001, .009] | 1.5% |
|  | General fractional anisotropy | −.110 (.022)^***^ | .011 (.022) | −.116 (.021)^***^ | .001 [7.83×10^-05^, .007] | 1.0% |
|  | General orientation dispersion | .283 (.022)^***^ | .022 (.025) | −.124 (.022)^***^ | .006 [.0002, .019] | 4.7% |

*Note:* All *β*s standardized. *β*_mediation_, the ‘indirect path’ from Sex to RT via the mediator, is with bootstrapped 95% confidence interval (1,000 iterations). RT = Reaction time, reversed such that higher scores indicate better performance. **p* < .05; ***p* < .01; ****p* < .001. *β_sex-RT_* path before inclusion of the mediator for first random half: *β* = .125, SE = .020, *p* < .001; for second random half: *β* = .091, SE = .020, *p* < .001. Model fit statistics for general fractional anisotropy in first random half: RMSEA = .087, CFI = .944, TLI = .922, SRMR = .047; second random half: RMSEA = .083, CFI = .949, TLI = .930, SRMR = .044. For comparative fit index in first random half: RMSEA = .082, CFI = .899, TLI = .856, SRMR = .059; second random half: RMSEA = .085, CFI = .892, TLI = .846, SRMR = .058.

*Table S12*. Percentage mediation of the sex-cognitive relation for each brain subregion selected by the LASSO model, for verbal-numerical reasoning and reaction time.

| Cognitive measure | Brain measure | Brain subregion | Mediation% |
| --- | --- | --- | --- |
| Verbal-numerical reasoning | Volume | Cuneus | 0.9% |
|  |  | Lateral orbitofrontal | 6.9% |
|  |  | Paracentral | 1.2% |
|  |  | Precentral | 6.1% |
|  |  | Precuneus | 7.9% |
|  |  | Superior temporal | 29.1% |
|  |  | Insula | 8.1% |
|  | Surface area | Entorhinal | 4.2% |
|  |  | Inferior temporal | 4.0% |
|  |  | Lateral orbitofrontal | 4.5% |
|  |  | Pars opercularis | 6.4% |
|  |  | Pars orbitalis | 1.6% |
|  |  | Pars triangularis | 3.2% |
|  |  | Pericalcarine | 4.6% |
|  |  | Posterior cingulate | 0.1% |
|  |  | Rostral middle frontal | 16.6% |
|  |  | Superior frontal | 2.8% |
|  |  | Superior temporal | 18.4% |
|  |  | Frontal pole | 0.4% |
|  |  | Insula | 4.7% |
|  | Thickness | Caudal anterior cingulate | 3.7% |
|  |  | Isthmus | 1.7% |
|  |  | Lateral occipital | 2.9% |
|  |  | Lateral orbitofrontal | 0.9% |
|  |  | Precentral | 3.0% |
|  |  | Rostral anterior cingulate | 0.7% |
|  |  | Supramarginal | 5.0% |
|  |  | Insula | 3.1% |
| Reaction time | Volume | Caudal anterior cingulate | 1.7% |
|  |  | Parahippocampal | 0.5% |
|  |  | Parstriangularis | 4.3% |
|  |  | Pericalcarine | 3.0% |
|  |  | Rostral middle frontal | 1.5% |
|  |  | Superior frontal | 2.6% |
|  |  | Frontal pole | 7.3% |
|  |  | Insula | 2.6% |
|  | Surface area | Caudal anterior cingulate | 4.4% |
|  |  | Caudal middle frontal | 2.8% |
|  |  | Isthmus | 5.1% |
|  |  | Lateral occipital | 4.3% |
|  |  | Pars triangularis | 1.2% |
|  |  | Perical carine | 3.4% |
|  |  | Superior parietal | 1.8% |
|  |  | Superior temporal | 0.1% |
|  |  | Temporal pole | 8.9% |
|  | Thickness | Parahippocampal | 0.6% |

*Table S13.* Peak regions of concatenated spatial maps of weighted degree from the resting-state fMRI analysis. Spatial clusters larger than 50 voxels were selected. The table shows the peaks of the spatial clusters.

| Sex association | Coordinate | Region (AAL template) | Hemisphere | Node | Group_ICA Cluster |
| --- | --- | --- | --- | --- | --- |
| Female > male | 12, −8, −38 | Crus II of cerebellar hemisphere | Right | 5,17,24 | DMN, AN/ECN |
|  | −8, −84, −36 | Crus II of cerebellar hemisphere | Left | 5,17,24,13 | DMN, AN/ECN |
|  | 58, −4, −16 | Medial temporal gyrus | Right | 10,34,42,52 | AN/ECN |
|  | −10, −58, 14 | Precuneus | Left | 10,11,36,48 | DMN, AN/ECN |
|  | 36, 4, 12 | Insula | Left | 13,19 | DMN, DMN' |
|  | −36, −18, 20 | Insula | Right | 19 | DMN |
|  | 52, −60, −8 | Inferior temporal gyrus | Right | 5,11,17,19,42,43 | DMN, AN/ECN |
|  | 58, 0, 4 | Superior temporal gyrus | Right | 10,52,53 | AN/ECN |
|  | −8, 2, 8 | Caudate | Left | 39 | AN/ECN |
|  | 22, 28, 44 | Medial frontal gyrus | Right | 13,24,34,48 | DMN, AN/ECN |
| Male > female | −14, −60, −22 | Lobule VI of cerebellar hemisphere | Left | 18 | AN/ECN |
|  | 18, -62, −22 | Lobule VI of cerebellar hemisphere | Right | 6,18 | SMN, ECN |
|  | 28, 58, 16 | Superior frontal gyrus | Right | 26,27,32,41, 50 | DMN, SMN |
|  | −22, 48, −12 | Superior frontal gyrus, orbital part | Left | 26, 32 | SMN, AN/ECN |
|  | 48, −8, 30 | Postcentral gyrus | Right | 20, 27, 30, 35 | SMN |
|  | 42, −64, 4 | Medial temporal gyrus | Right | 23, 27, 49 | SMN, DMN |
|  | −40, −66, 6 | Medial occipital gyrus | Left | 4 | AN/ECN |
|  | −30, 58, 14 | Medial prefrontal gyrus | Left | 8,12 | VN, DMN' |
|  | −12, −20, 0 | Thalamus | Left | 47 | SN |
|  | 6, 38, 28 | Midcingulate | Right | 26 | AN/ECN |
|  | 12, −20, 0 | Thalamus | Right | 47 | SN |
|  | −24, −76, 34 | Superior occipital gyrus | Left | 6 | SMN |
|  | 30, −68, 30 | Medial occipital gyrus | Right | 18 | AN/ECN |

*Note:* DMN = Default Mode Network; DMN’ = Extended Default Mode Network; SMN = Sensorimotor Network; AN/ECN = Attention Network/Executive Control Network; VN = Visual Network; SN = Salience Network.

*Table S14.* Results from the resting-state fMRI analysis. Connection strength values from partial and full correlations, and weighted degree values for each node.

See Excel spreadsheet available at the Open Science Framework. Direct link: <https://osf.io/rt7es/>

**Supplemental Figures**

*Figure S1.* Labelled subcortical regions examined in the study.

*Figure S2a.* Shift functions for each of the overall and subcortical variables. The central, darkest line on each distribution is the median, and the other lines demarcate the deciles of each distribution. The values refer to the number of units (cm^3^) that the labelled part of the female (upper) distribution would have to be shifted to match the male (lower) distribution. Note that the figures appear across multiple pages.


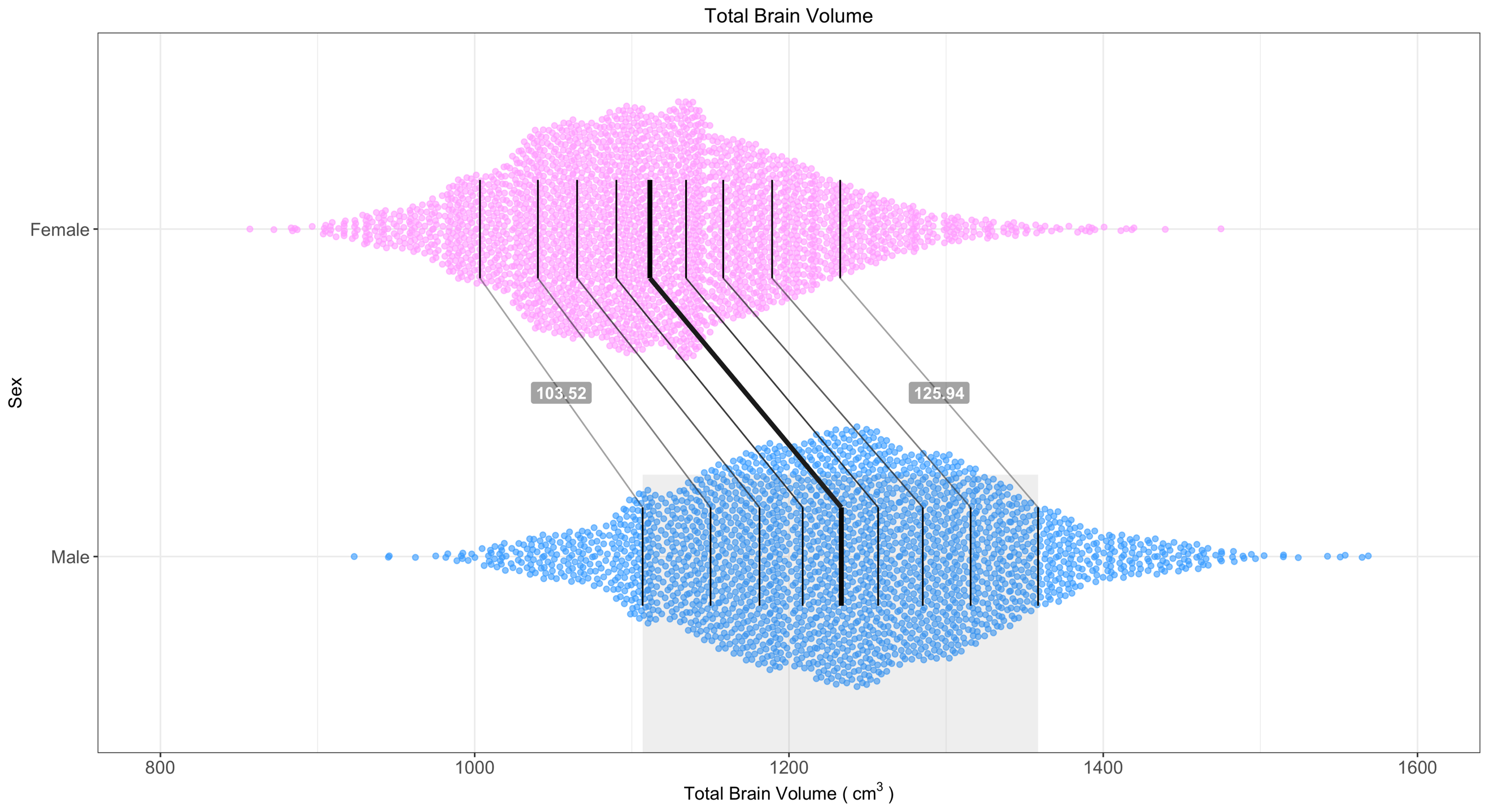


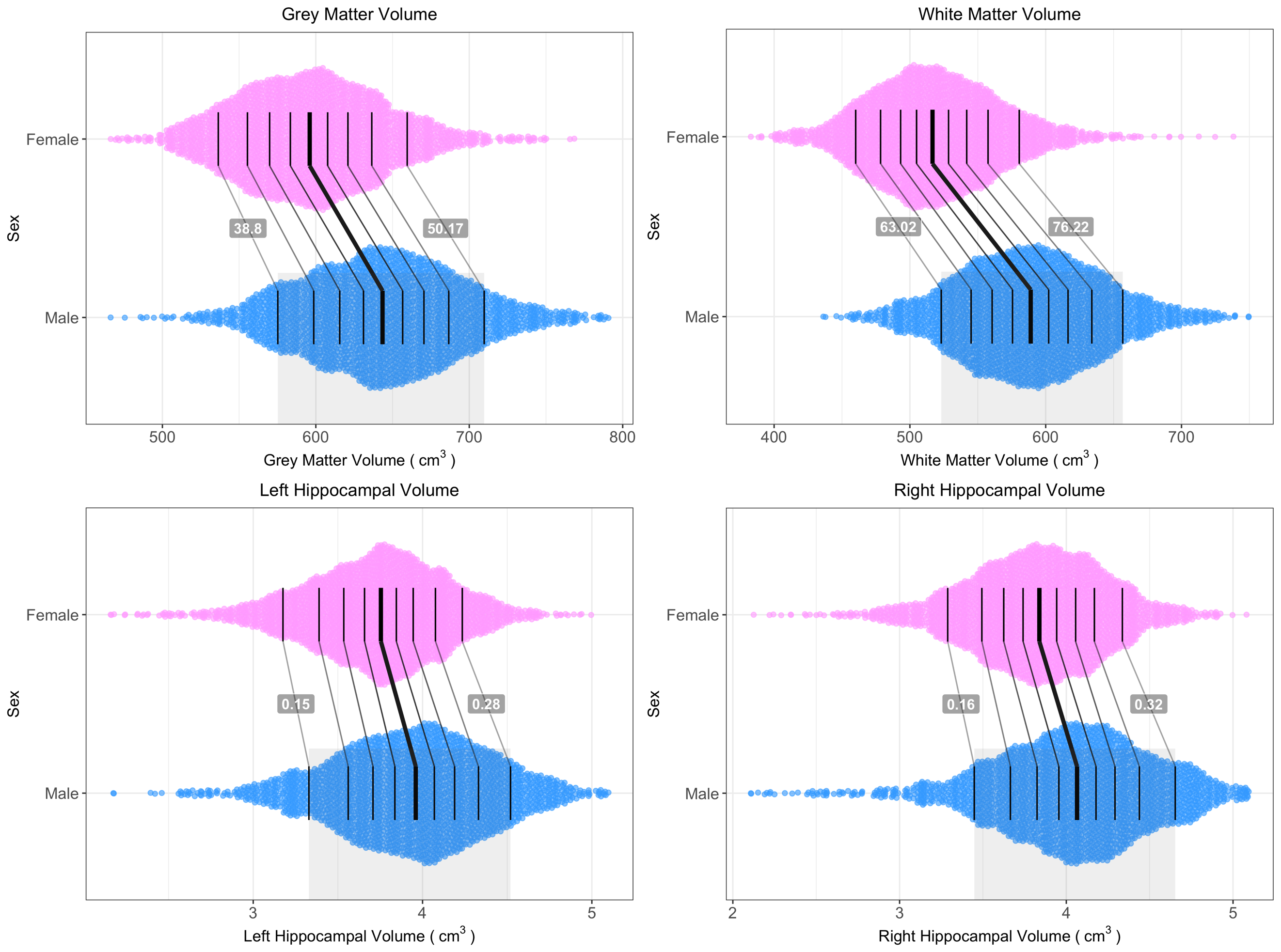


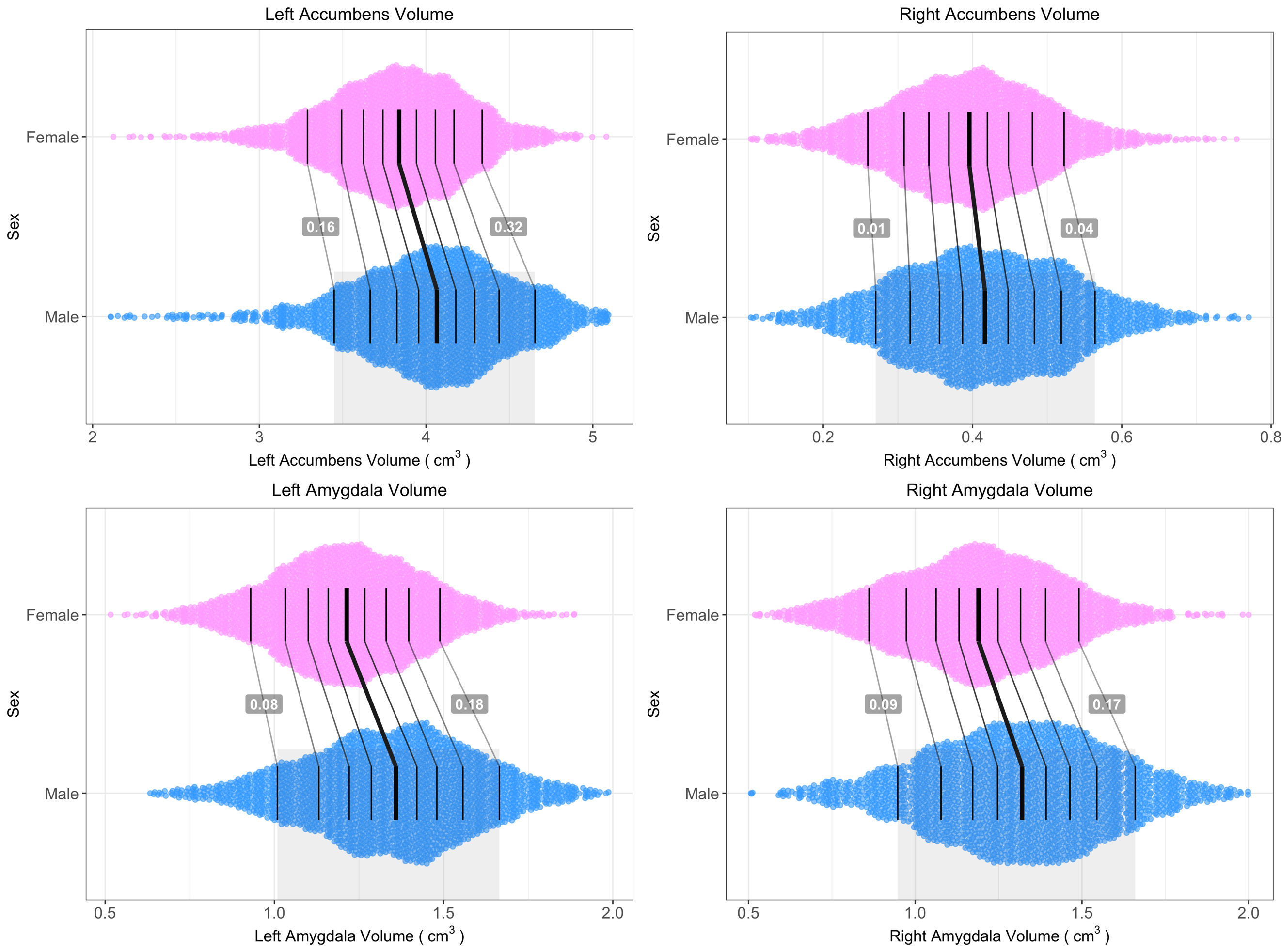


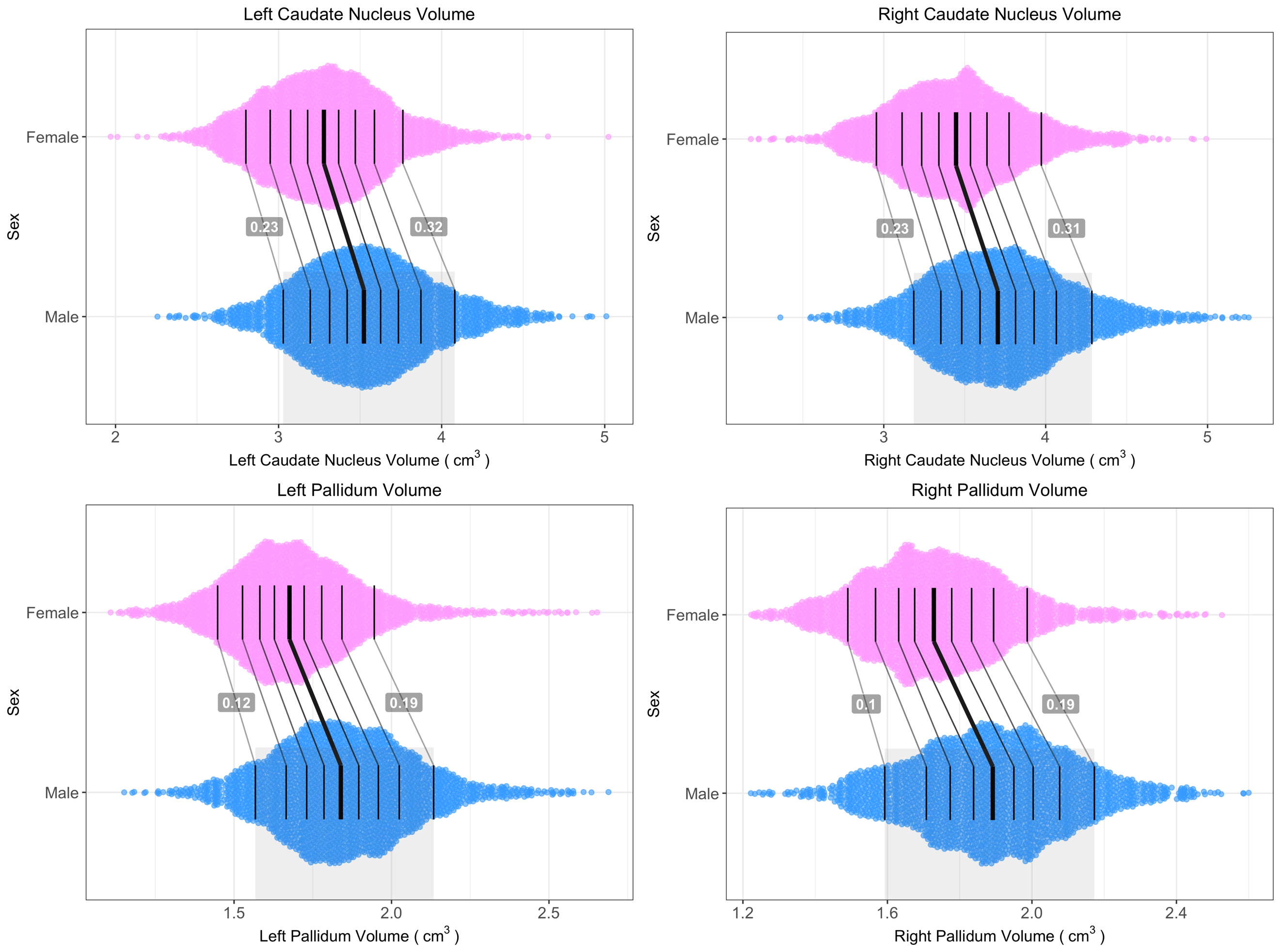


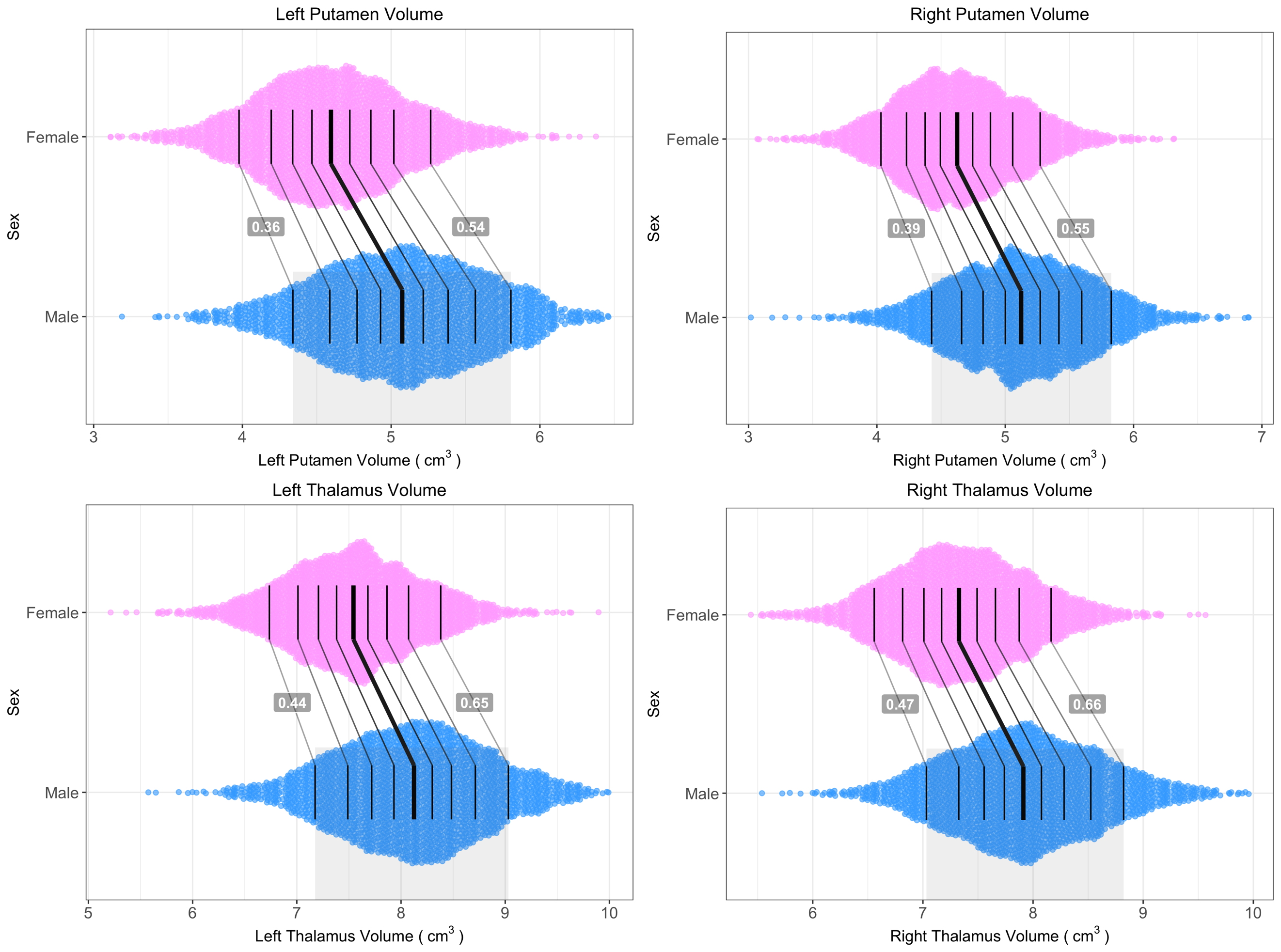


*Figure S2b.* Shift functions for each of the subcortical variables, adjusted for total brain volume (TBV). The central, darkest line on each distribution is the median, and the other lines demarcate the deciles of each distribution. The values refer to the number of standardized units that the labelled part of the female (upper) distribution would have to be shifted to match the male (lower) distribution. Note that the figures appear across multiple pages.

*
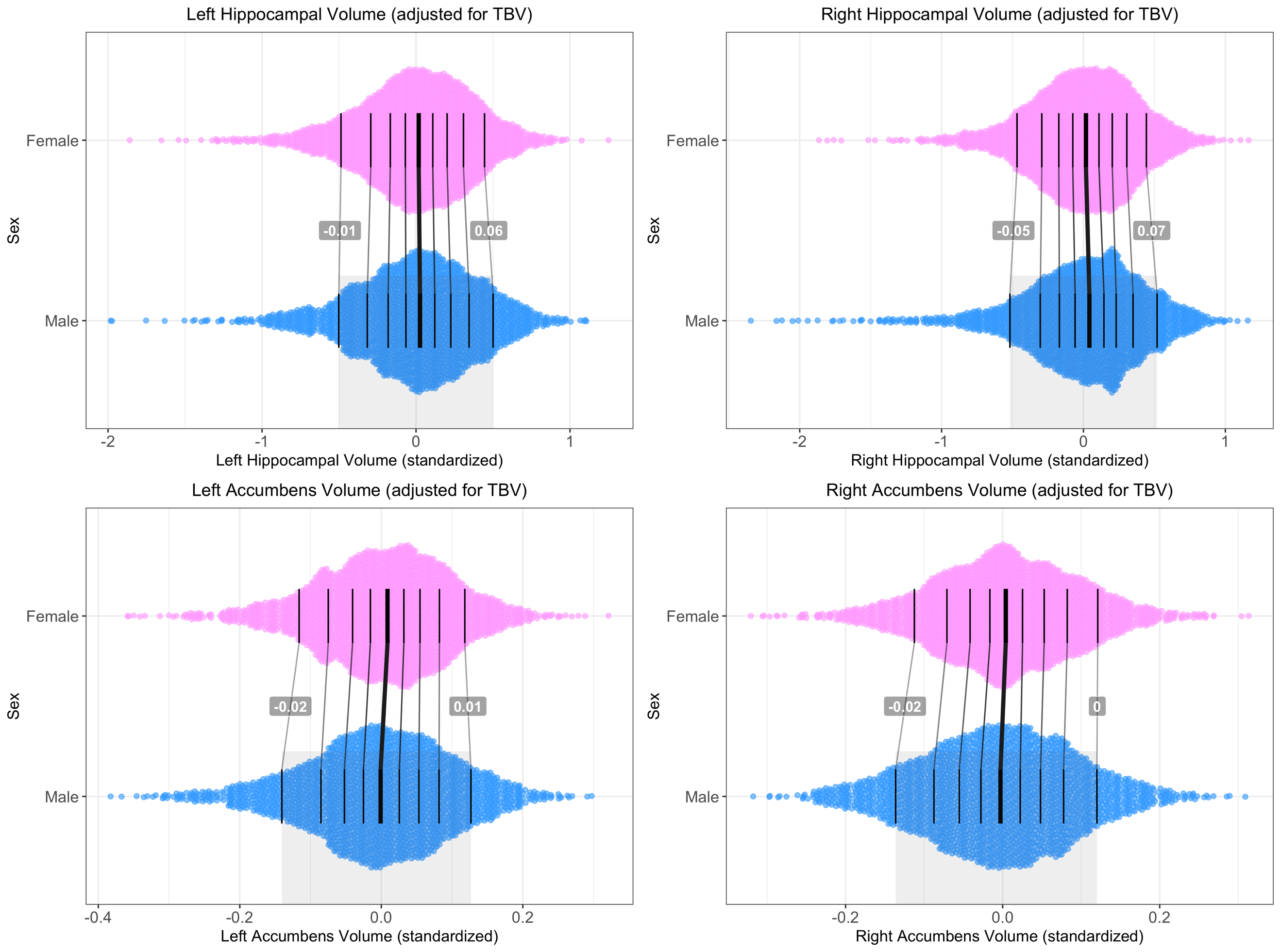
*

*
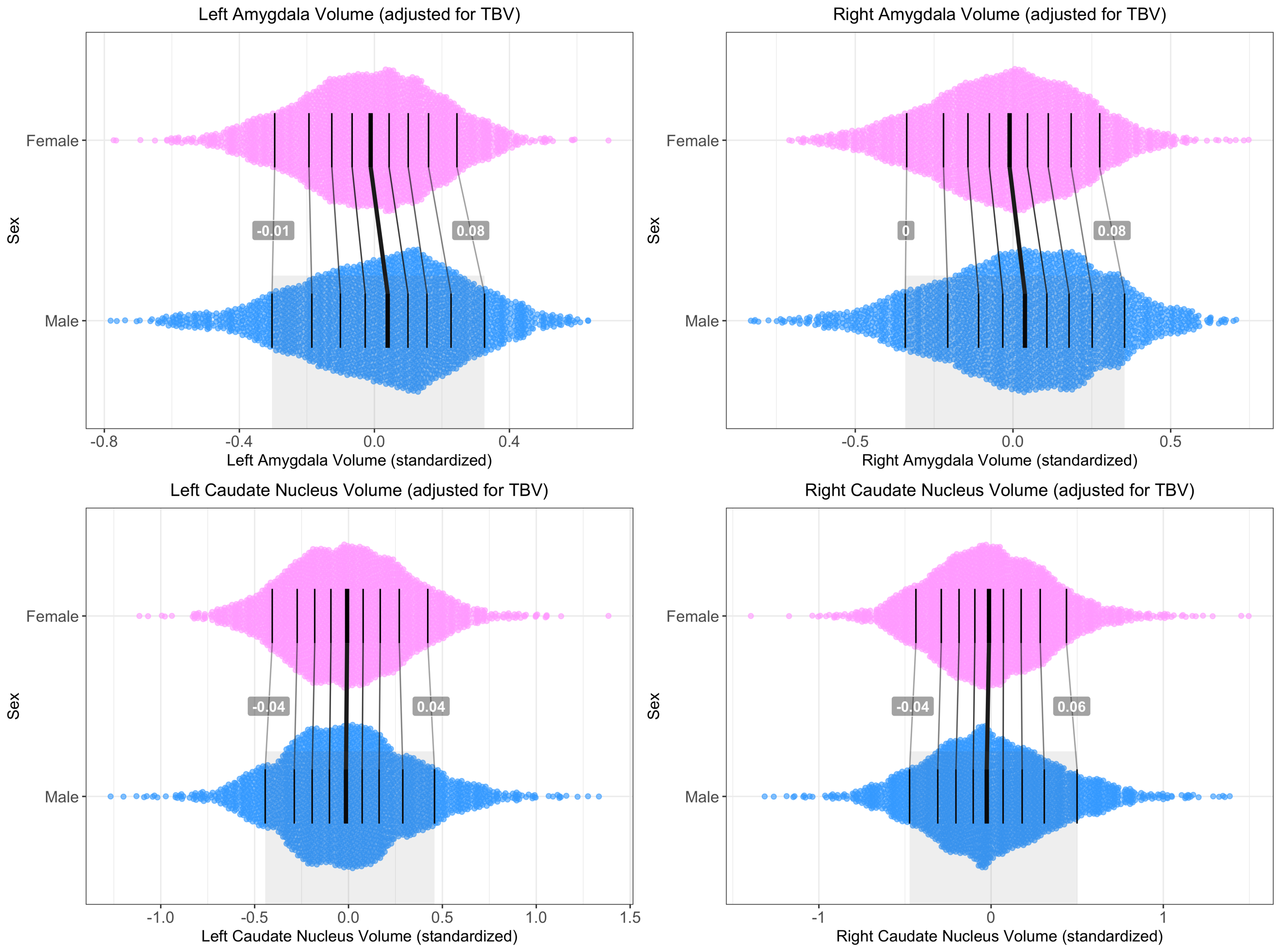
*

*
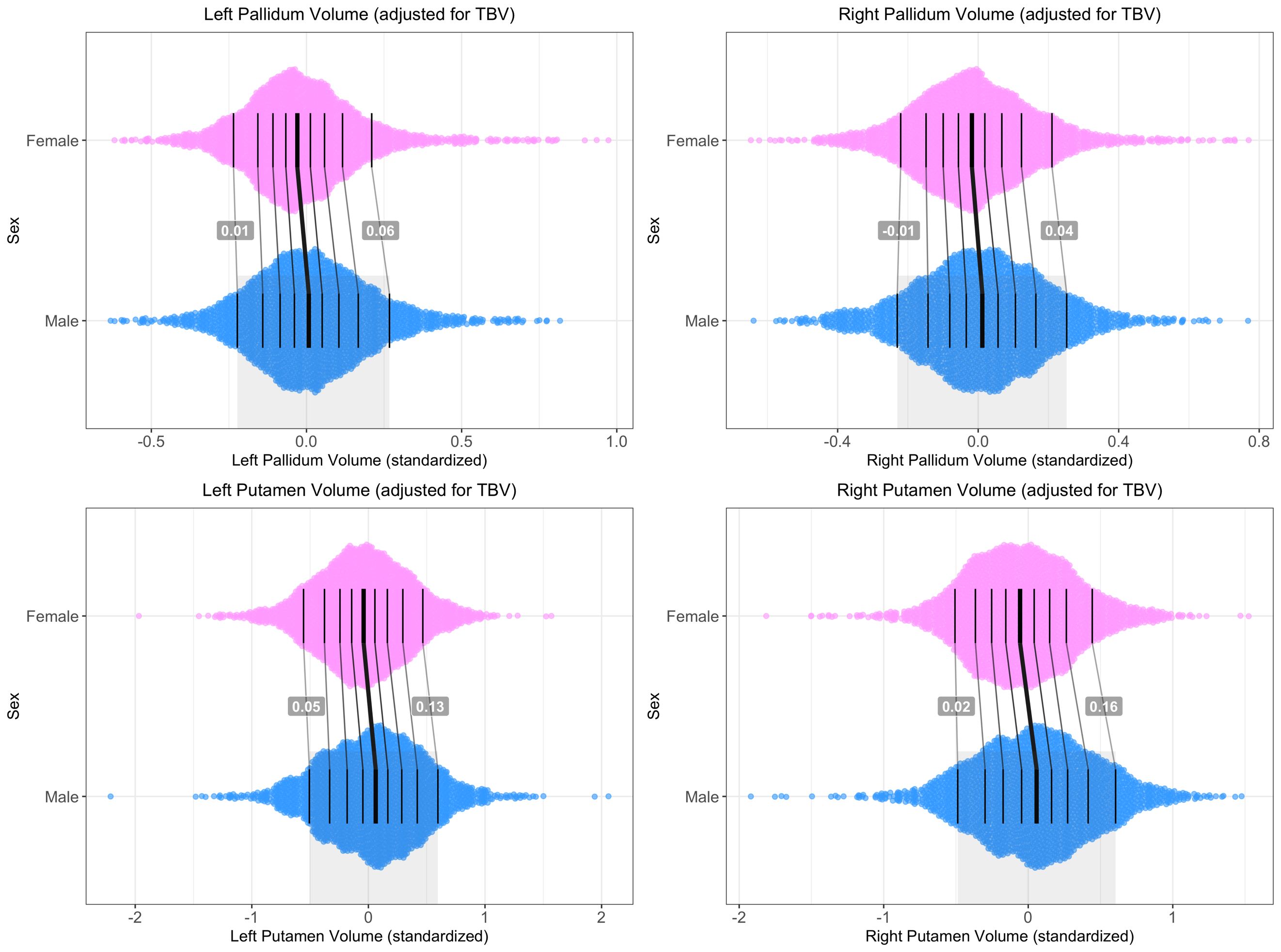
*

*
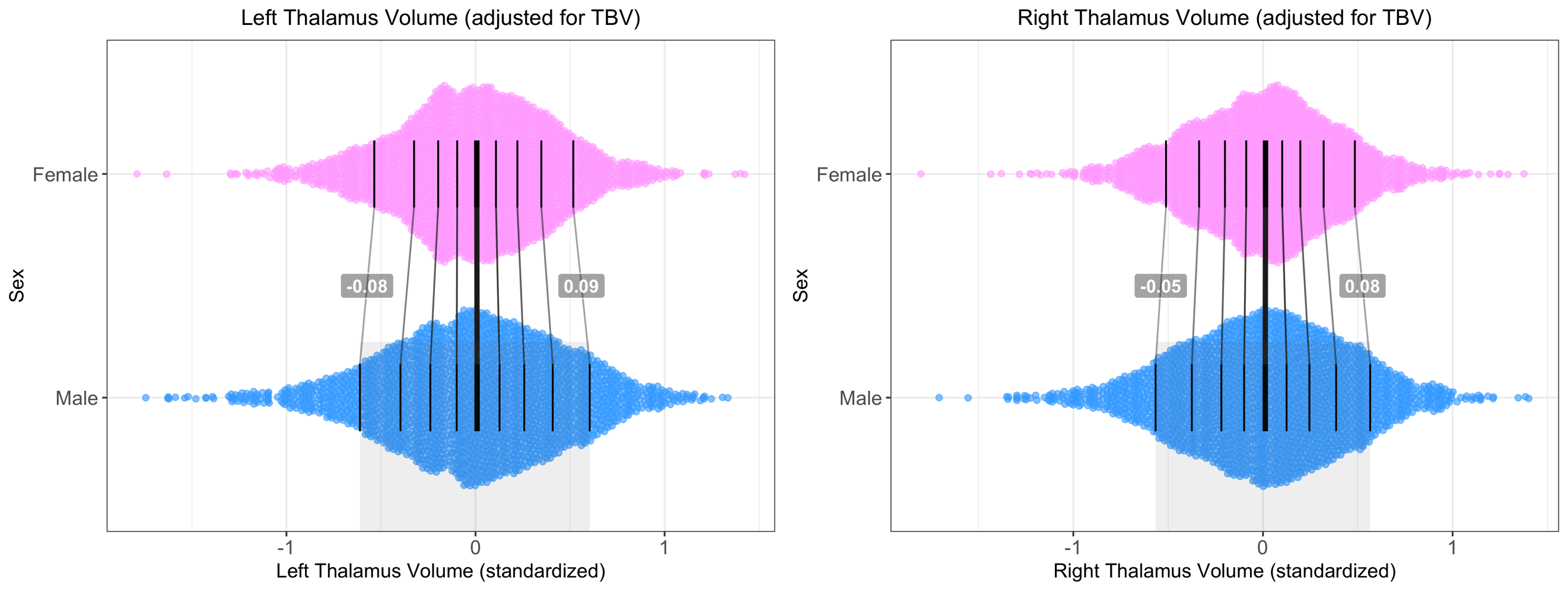
*

*Figure S3.* Labelled regions of interest from the Desikan-Killiany neuroanatomical atlas.

**

*Figure S4*. Labelled white matter tracts of interest.

*Figure S5.* Sex differences in variance across the brain subregions in Volume, Surface Area, and Cortical Thickness, adjusting for total brain volume, total surface area, or mean cortical thickness, respectively. See Figure S3 for a brain region atlas.

*Figure S6.* Correlations between the mean difference (Cohen’s *d*) and the variance difference (Variance Ratio) across all 68 regions of the cortex, for volume, surface area, and cortical thickness. Values are unadjusted. For reference, lower positions on both axes indicate greater male variability/higher male means. The shaded area around each line is the 95% confidence region.


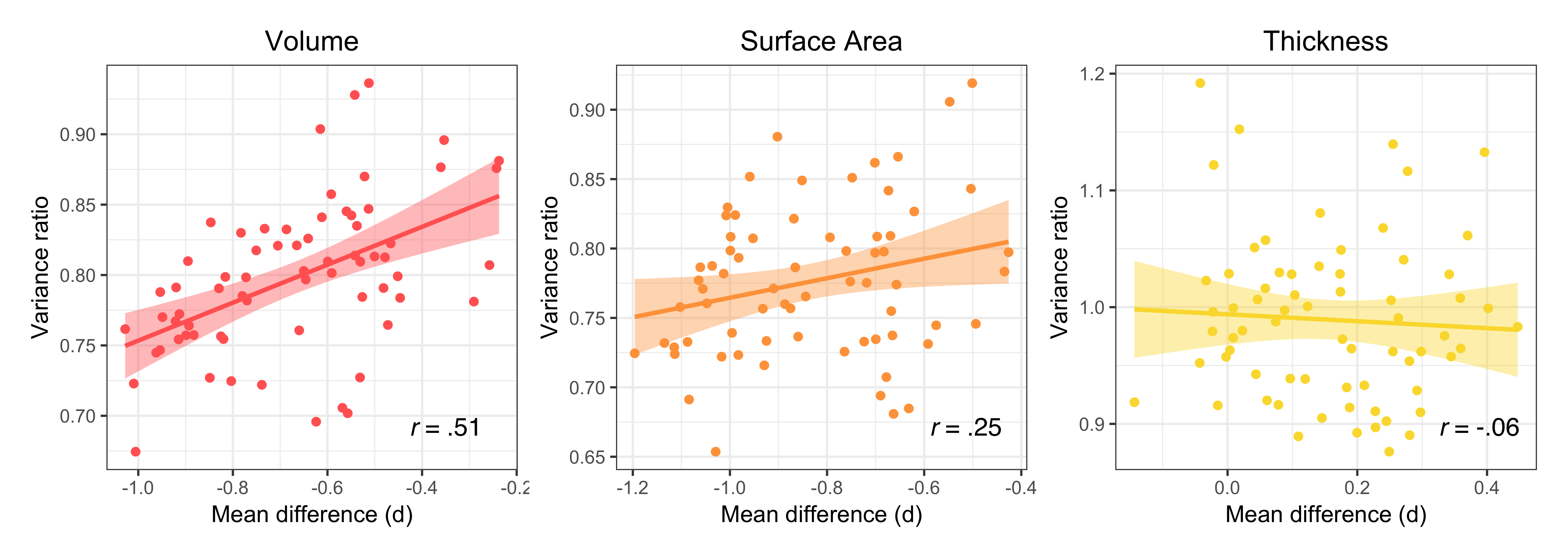


*Figure S7.* Correlations between the mean difference (Cohen’s *d*) and the variance difference (Variance Ratio) across all 68 regions of the cortex, for volume, surface area, and cortical thickness, adjusted for total brain volume. For reference, lower positions on both axes indicate greater male variability/higher male means. The shaded area around each line is the 95% confidence region.


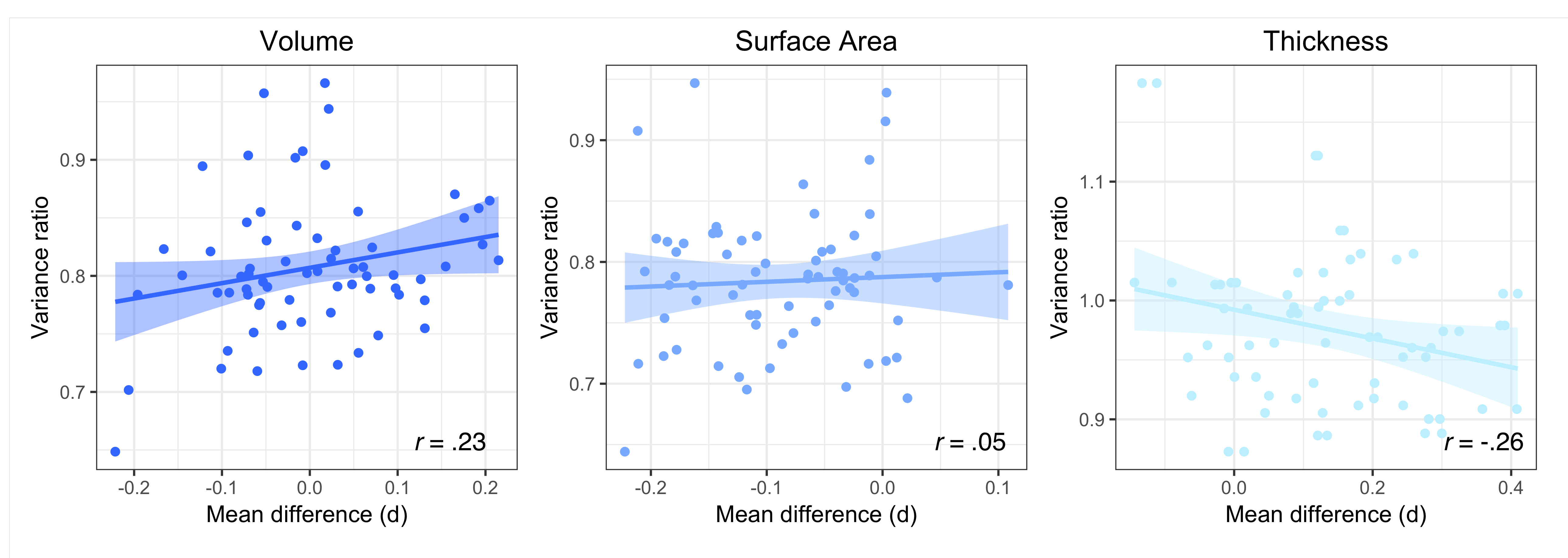


*Figure S8.* Sex differences in means for brain volume, surface area, and cortical thickness using a vertex-wise analysis.

*Figure S9.* Sex differences in variances for brain volume, surface area, and cortical thickness using a vertex-wise analysis.

**

*Figure S10*. Sex differences in variance in diffusion-tensor measures A) fractional anisotropy and B) orientation dispersion across 22 white matter tracts. See Figure S4 for tract atlas.

*Figure S11.* Path diagram for the mediation model. The direct path from sex to cognitive ability (either verbal-numerical reasoning or reaction time) is hypothesized to be mediated by the indirect path via the brain measure (total brain volume, grey matter volume, white matter volume, total cortical surface area, mean cortical thickness, general fractional anisotropy, or general orientation dispersion). Results, with estimates for each of the paths shown, are provided in Tables S10 and S11 for verbal-numerical reasoning and reaction time, respectively.

*Figure S12.* Performance of each brain-level predictor (volume, surface area, and cortical thickness) in a LASSO model predicting (A) verbal-numerical reasoning and (B) reaction time test performance. For each brain measure, the left graph shows the cross-validation process for the shrinkage/penalty parameter: the mean-squared error of the model at each level of the shrinkage parameter, as shown on the x-axis increasing from left to right. The lowest point, marked with the leftmost vertical dotted line, is the shrinkage value chosen for variable selection (λ_min_). This value is also shown by a dotted line in the right (coefficient path) graph, which illustrates the coefficients of each predictor as the shrinkage parameter increases. The values at the top of each plot refer to the number of predictor variables remaining in the model for the corresponding level of the shrinkage parameter. In the coefficient path graphs, the numbers on each path refer to the corresponding brain measure of the following (bilaterally-averaged) areas: 1: banks of superior temporal; 2: caudal anterior cingulate; 3: caudal middle frontal; 4: cuneus; 5: entorhinal; 6: fusiform; 7: inferior parietal; 8: inferior temporal; 9: isthmus; 10: lateral occipital; 11: lateral orbitofrontal; 12: lingual; 13: medial orbitofrontal; 14: middle temporal; 15: parahippocampal; 16: paracentral; 17: pars opercularis; 18: pars orbitalis; 19: pars triangularis; 20: perical carine; 21: postcentral; 22: posterior cingulate; 23: precentral; 24: precuneus; 25: rostral anterior cingulate; 26: rostral middle frontal; 27: superior frontal; 28: superior parietal; 29: superior temporal; 30: supramarginal; 31: frontal pole; 32: temporal pole; 33: transverse temporal; 34: insula. Note that the figures appear across multiple pages.

*Figure S13.* Resting-state functional connections showing significant relations with sex. Blue values indicate stronger associations in males; red values indicate stronger associations in females. From bottom-left to top-right, the dark squares are the connections within the visual network (blue numbers in Figure 5A in the main document), the sensorimotor network (yellow/orange in Figure 5A), the default mode network (red in Figure 5A), the salience/executive control network (purple in Figure 5A), and the dorsal attention network (green in Figure 5A).


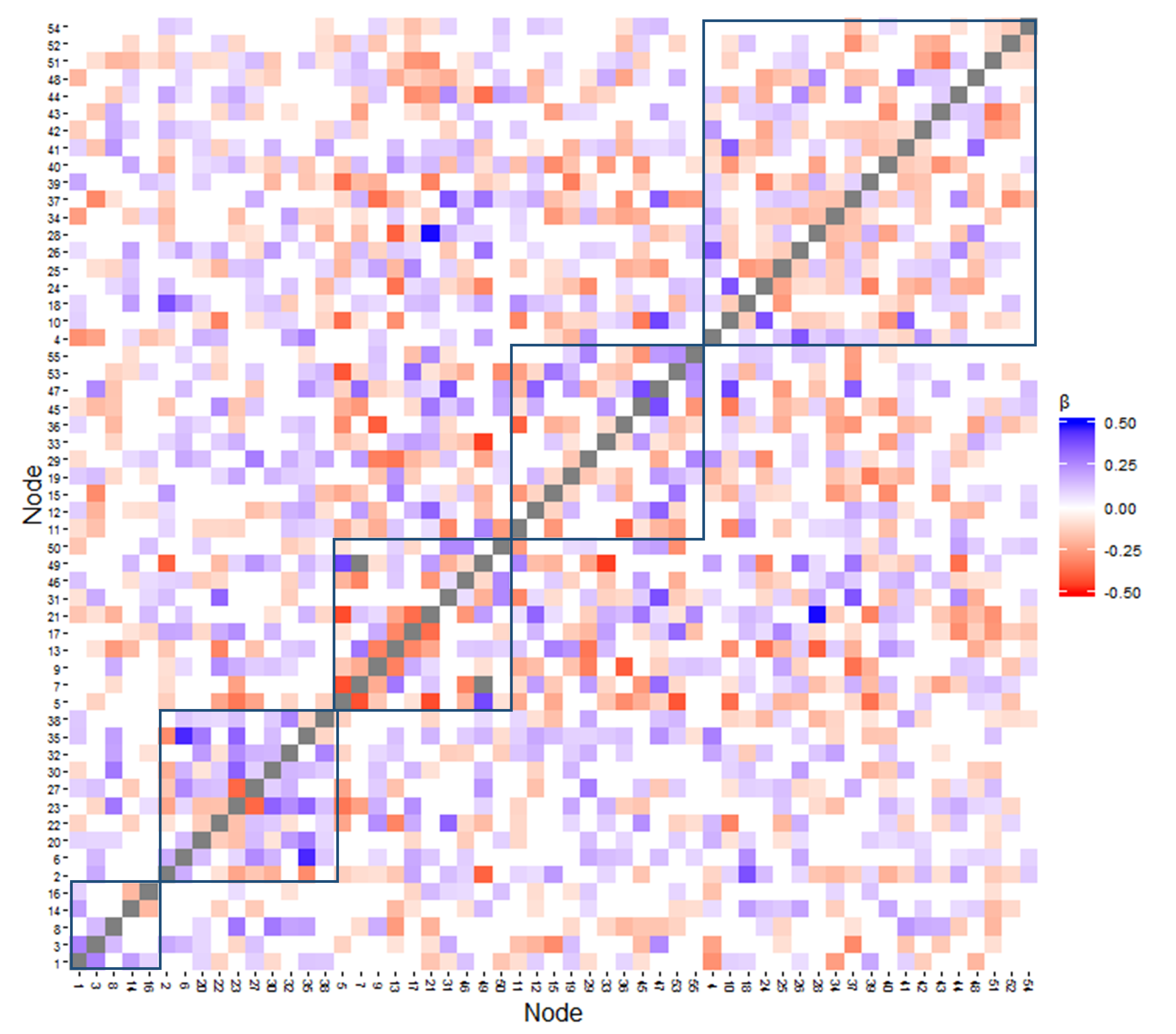

Supplement: Supplementary Data [file bhy109_supplementarymaterials.zip › bhy109RitchieUKBSexDiffsSUPPLEMENT30Mar18.docx]
